# Vegetable Oil‐Based Materials for Drug Delivery Systems and Wound Dressings

**DOI:** 10.1002/mabi.202500486

**Published:** 2025-11-27

**Authors:** Lucas M. Favre, Nicolas Masurier, Anne Aubert‐Pouëssel

**Affiliations:** ^1^ ICGM Univ Montpellier, CNRS, ENSCM Montpellier France; ^2^ IBMM Univ Montpellier, CNRS, ENSCM Montpellier France

**Keywords:** biocompatibility, biopolymers, drug delivery, mechanical properties, triglyceride chemical functionalization, vegetable oils, wound dressing

## Abstract

Vegetable oils are natural and renewable resources, mostly composed of triglycerides (fatty acid esters of glycerol). These molecules possess multiple reactive sites, which can be used for chemical functionalization to form epoxides, hydroxyls, and cyclic carbonates. Thanks to these added functions, polymerization can take place in order to form vegetable oil‐based materials, such as polyesters, polyurethanes, or hybrid materials. The development of vegetable oil‐based polymers has provided access to new materials with properties such as flexibility, biocompatibility, and biodegradability. Thus, these characteristics make them particularly well‐suited for biomedical applications. In this review, we are focusing on vegetable oil‐based materials developed as drug delivery systems and wound dressings.

## Graphical abstract

Renewable resources like vegetable oils are increasingly explored for biomaterial production in biomedical applications. Their monoglycerides and triglycerides inherently possess functions or can be chemically modified to introduce functional groups suitable for polymerization, yielding biomaterials with desirable properties such as flexibility, biocompatibility, and biodegradability for medical use, such as drug delivery systems and wound dressings.

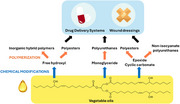

**Abbreviations**
5‐FU: 5‐fluorouracil
APS: (3‐aminopropyl)trimethoxysilane
ASBO: azide‐functionalized Soybean Oil
BDA: 1,4‐butanediamine
CA: citric acid
CHM: cyclohexanobis(methylamine)
CO: castor oil
CO‐p(MCS): castor oil‐sebacic acid‐mannitol‐citric acid
CSA: camphorsulfonic acid
CSBO: carbonated soybean oil
DBTDL: dibutyltin dilaurate
DDS: drug delivery systems
ECH: epichlorohydrin
ECO: epoxidized castor oil
ELA: epoxidized linseed oil
EQ: *echium* oil
ESBO: epoxidized soybean oil
GTEAC: glycidyltriethylammonium chloride
HCC: hydroxyl containing component
HFF: human foreskin fibroblasts
HMDI: hexamethylene diisocyanate
HPMC: hydroxypropyl methylcellulose
HTPB: hydroxyl‐terminated polybutadiene
ICO: silylated castor oil
IMC: indomethacin
IPDI: isophorone diisocyanate
IPTES: isocyanatopropyl triethoxysilane
LA: D,L‐lactic acid
LA‐EG: ethylene glycol‐lactic acid
LO: linseed oil
mCPBA: *m*‐chloroperoxybenzoic acid
MDI: methylene diphenyl diisocyanate
NIPU: non‐isocyanate polyurethane
NSO: nigella sativa oil
PBS: phosphate‐buffered saline
PCO: phosphorylated castor oil
PEG: polyethylene glycol
PLGA: poly(lactic‐co‐glycolic acid)
PONC: polymer‐oil nanostructured carriers
PU: polyurethane
PVA: poly(vinyl alcohol)
PVP: poly(vinylpyrrolidone)
QTSBO: 1,2,3‐triazolium‐functionalized soybean oil
RA: ricinoleic acid
RM: ricinoleic methyl ester
SAM: N‐stearoyl‐L‐alanine methyl ester
SBO: soybean oil
Si‐AT: trimethoxysilane‐terminated aniline tetramer
SMP: sorbitan monopalmitate
SMS: sorbitan monostearate
SnOct: tin(II) 2‐ethylhexanoate
TDI: toluene diisocyanate
TEPA: tetraethylenepentamine
TSBO: 1,2,3‐triazol functional soybean oil
UV: ultraviolet
VO: vegetable oil

## 1 Introduction

**FIGURE 1:**
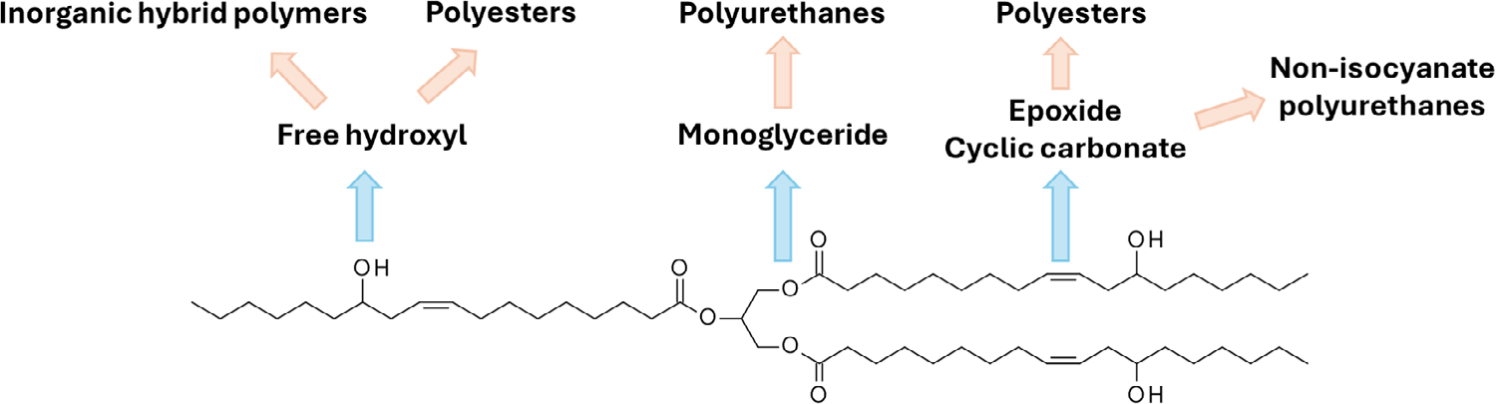
Strategies for polymer synthesis from the main triglyceride of castor oil: triricinolein (glycerol triricinoleate).

The development of polymeric materials for biomedical applications, such as tissue engineering, wound healing, or drug delivery, has been in great expansion in these last few years, due to advances in medical research, physiological knowledge, and technological innovations. Polymers with carbon backbones derived from fossil fuels offer a wide range of properties (biocompatibility, mechanical properties, biodegradability, etc.), that make them suitable for biomedical applications. However, in an age of global warming and environmental concerns, natural molecules from renewable resources, such as cellulose, starch, chitosan or vegetable oils (VOs), have emerged as attractive and sustainable alternatives for the production of biopolymers. Hence, polymers based on triglycerides from vegetable oils have been developed [1], improving not only their environmental footprint, but also providing original mechanical properties to the material, while ensuring biocompatibility and biodegradability [2]. Triglycerides are the main constituents of VOs, and contain multiple functional groups, such as esters, double bonds, and secondary alcohols (Figure 1) [3], that can undergo chemical modifications and be used as monomers for polymer synthesis. Moreover, the inherent lipophilic nature of these monomers can be exploited to solubilize lipophilic bioactive molecules, with the goal to confer therapeutic properties to the resulting polymeric materials.

Recent and insightful reviews have explored the potential of VO‐based polymers in biomedical applications [4, 5, 6, 7]. While these works provide valuable general overviews, the present review offers a more focused perspective by specifically addressing materials derived from VOs for applications in drug delivery and wound healing.

## 2 Vegetable Oils and Their Use in Drug Delivery and Wound Dressing Applications

### 2.1 Brief Introduction to Vegetable Oils

**FIGURE 2:**
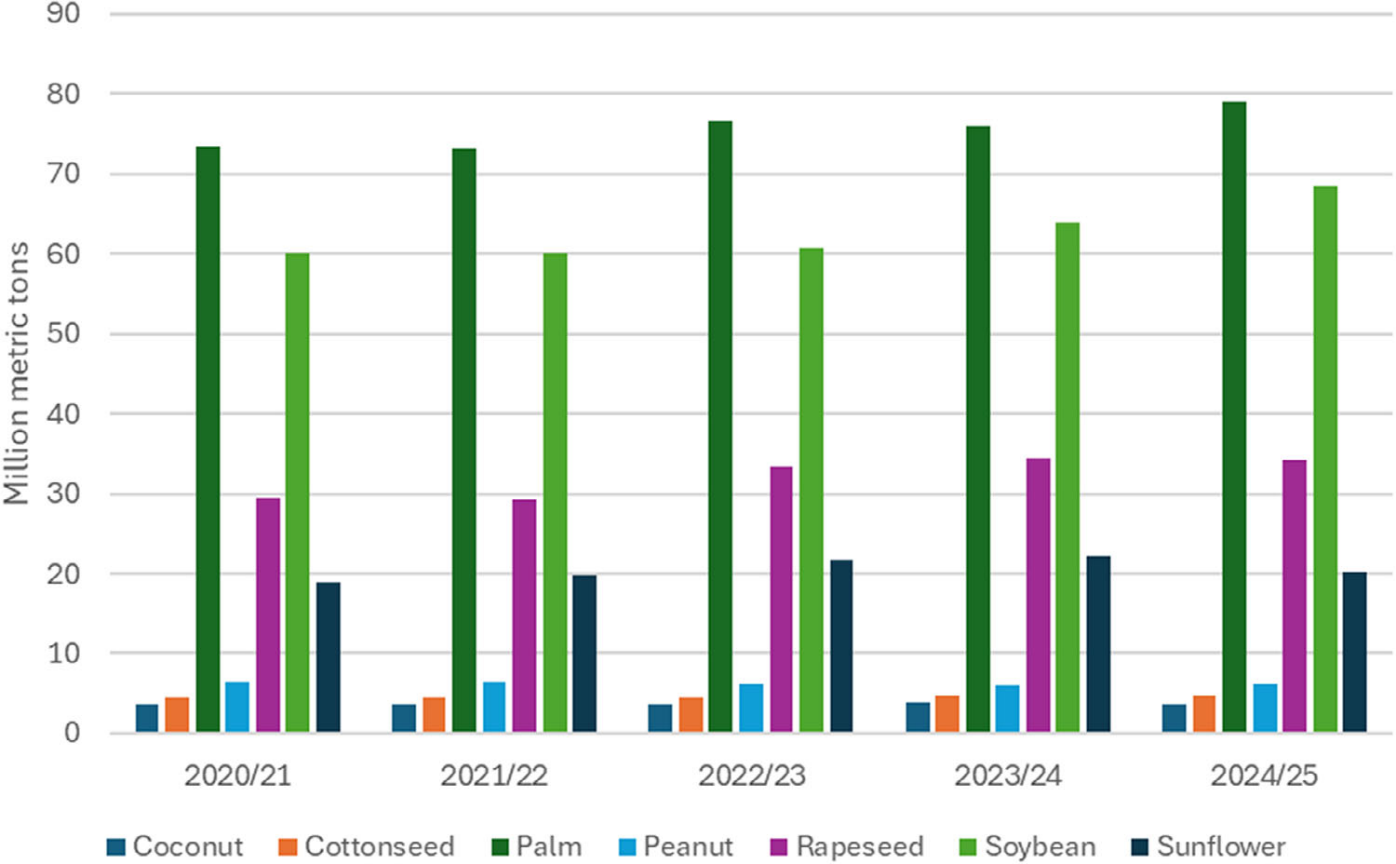
Global production of edible VOs from 2020 to 2025, according to data from USDA.

Vegetable oils are natural and renewable resources produced from land plants. They are produced in huge quantities, especially the edible VOs (coconut, cottonseed, palm, peanut, rapeseed, soybean, or sunflower), and are widely consumed all over the globe, as reported by the United States Department of Agriculture (USDA) in September 2025 (https://apps.fas.usda.gov/psdonline/circulars/oilseeds.pdf) and illustrated in Figure 2. Among them, palm oil is the most produced VO, reaching 79 million tons in 2024/2025, followed closely by soybean at 68 million tons. On the other hand, non‐edible oils (castor, linseed, neem, jojoba) are generally used for industrial purposes, notably as lubricants for the car industry [8]. or feedstocks for biofuel production [9].

Vegetable oils are composed of complex mixtures of multiple triglycerides, which differ between each plant species (Table 1). Triglycerides are fatty acid esters of glycerol, and their chemical structures vary from saturated (stearic) to unsaturated chains (palmitoleic, oleic, linoleic, linolenic, α‐eleostearic…), and may contain multiple double bonds, as well as chemical functions such as hydroxyls (ricinoleic) or epoxides (vernolic) (Table 2). They are classified according to their chain length and number of unsaturation: for example, stearic acid is classified as C18:0 (chain length of 18 carbons, no unsaturation), and linolenic acid is a C18:3 (chain length of 18 carbons with 3 double bonds).

**TABLE 1:** Degree of unsaturation and fatty acid compositions of natural VOs [4, 10].

	Fatty acid composition (%)					
VOs	Double bond	C16:0	C18:0	C18:1	C18:2	C18:3
Castor	3.0	1.5	0.5	5.0	4.0	0.5
Coconut	—	9.8	3.0	6.9	2.2	—
Corn	4.5	10.9	2.0	25.4	59.6	1.2
Cottonseed	3.9	21.6	2.6	18.6	54.4	0.7
Linseed	6.6	5.5	3.5	19.1	15.3	56.6
Olive	2.8	13.7	2.5	71.1	10.0	0.6
Palm	1.7	42.8	4.2	40.5	10.1	—
Palm kernel	—	8.8	2.4	13.6	1.1	—
Peanut	3.4	11.4	2.4	48.3	31.9	—
Rapeseed	3.8	4.0	2.0	56.0	26.0	10.0
Sesame	3.9	9	6	41	43	1
Soybean	4.6	11.0	4.0	23.4	53.3	7.8
Sunflower	4.7	5.2	2.7	37.2	53.8	1.0

**TABLE 2:** Structures of common fatty acids.

C16:0	Palmitic acid	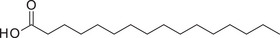
C18:0	Stearic acid	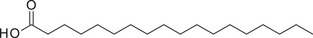
C18:1	Oleic acid	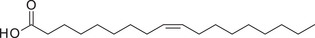
	Ricinoleic acid	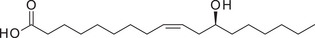
	Vernolic acid	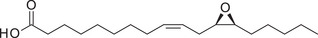
	Petroselenic acid	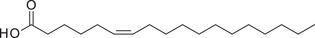
C18:2	Linoleic acid	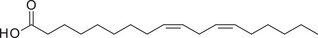
C18:3	Linolenic acid	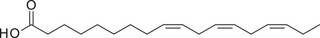
	Calendic acid	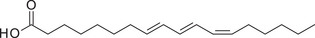
	α‐eleostearic acid	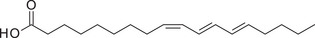

Thanks to their natural abundance, renewability, and ease of chemical modifications, VOs have attracted significant research interest as raw materials for polymer synthesis. As a result, VO‐based polyesters and polyurethanes have been developed [11], offering a more sustainable alternative to fossil fuel‐derived polymers. Moreover, their biodegradability and biocompatibility are particularly promising for biomedical applications.

### 2.2 Needs in Drug Delivery Systems (DDS)

Among biomedical applications, drug delivery systems (DDS) represent a field with demanding requirements and promising potential for VO‐based materials. Monolithic (film, gel) or particulate DDS are able to load therapeutic compounds, whether derived from conventional medicinal chemistry or biotechnologies, and to release them in a controlled manner, either progressively or triggered [12]. DDS can enhance the bioavailability of therapeutic compounds, ensure controlled drug release, allow targeted delivery to specific sites, reduce adverse effects, and overall improve patient compliance in many therapeutic fields, particularly in analgesia and oncology.

To meet these objectives, DDS must be biocompatible, sterile, degradable, and easy to produce on an industrial scale, regardless of their administration route. Cytocompatibility is typically assessed in vitro by fibroblast proliferation assays, and in vivo biocompatibility is evaluated after subcutaneous implantation, observing signs of inflammation. Drug release studies are essential studies carried out first in vitro to select the best formulations and then in vivo. A wide variety of materials have been explored for the development of DDS, including synthetic or natural polymers, lipids, and minerals. Each brings its own specific characteristics, and VO‐based polymers offer unique properties. VOs have the advantage of being readily available in large quantities, can be easily chemically modified, and allow simple formulation processes such as emulsification. They are able to solubilize highly lipophilic active ingredients, physico‐chemical properties that are frequently observed for molecules under development. Such polymers can be biodegraded in a physiological environment by lipase‐type enzymes, and their metabolites can be assimilated by the body. Many vegetable oils have long been used in pharmacy, and their biocompatibility is well established for various routes of administration, including injectable and topical applications. All these characteristics make them particularly relevant for drug delivery applications.

### 2.3 Needs in Wound Dressing Applications

Another important biomedical application of VO‐based materials lies in wound dressings, which also impose complex functional requirements. The wound healing process is multifaceted and occurs in three overlapping stages: inflammation, proliferation, and maturation. In the inflammation phase, platelets in the exposed blood aggregate to form a coagulation “plug”. This is followed by the action of phagocytes, which clear foreign substances, enabling fibroblast proliferation and collagen deposition. Finally, collagen fibers mature and remodel to form the scar [13].

Materials used in wound healing applications must cover the wound and protect it from infections and microorganisms, maintain moisture balance, and allow gas exchange while being biocompatible, elastic, and stay in place at the wound location. Mechanical performance is equally important. The elasticity of the material is measured with a tensile tester, which determines the Young's modulus, tensile strength, and elongation at break. A lower Young's modulus value indicates greater elasticity. The tensile strength is the maximum stress a material can withstand before breaking, and the elongation at break can be measured at that point. Higher values of tensile strength and elongation at break reflect good mechanical integrity and flexibility, essential for materials applied to dynamic wound sites.

Moisture management properties are evaluated through water uptake and surface hydrophilicity. Contact angle measurements using a goniometer determine the surface wettability. A polymer is considered to have a hydrophilic surface if the measured contact angle is lower than 90°. Water absorption capacity is measured by weighing the polymer before and after immersion in distilled water, following removal of excess water on the film surface.

Beyond wound coverage, the development of films with intrinsic antibacterial activity may be an interesting option, providing both a physical barrier and active protection against infections. Antibacterial efficacy can be evaluated using the zone of inhibition method, which involves measuring the clear area devoid of bacterial growth surrounding polymer disks placed on an agar plate inoculated with the studied bacterial strain. A bacterial viability assay can also be used, consisting of incubating polymer discs in wells containing bacterial suspension and subsequently quantifying the number of viable bacteria.

Materials used in wound dressing applications are varied: natural or synthetic polymers, and can be fabricated in multiple formats: films, foams, hydrogels, hydrocolloids, or membranes [13]. Vegetable oil‐based polymers offer interesting properties for wound dressing design. In addition to the properties already described above, they can easily be shaped into films, their hydrophobicity can be modulated to provide varying degrees of impermeability, and their mechanical properties can be tuned to adjust film flexibility. Finally, the biocompatibility of vegetable oils with the skin has been widely demonstrated, making these materials promising candidates for the development of wound dressings.

## 3 Modified Vegetable Oil‐Based Materials

To fulfill the functional requirements of drug delivery systems and wound dressings, triglycerides, the primary constituents of VOs, must contain reactive functional groups within their molecular structure to undergo polymerization. While some vegetable oils naturally contain such reactive moieties, their occurrence is limited. Therefore, the chemical functionalization of vegetable oils has become a crucial step in the development of bio‐based polymers for biomedical applications. Several strategies have been explored to introduce polymerizable functional groups into triglyceride structures. Among the commonly employed modifications are epoxidation, which introduces epoxide rings onto unsaturated fatty acid chains; hydroxylation, which converts double bonds into hydroxyl groups; and the formation of cyclic carbonates, offering reactive sites for ring‐opening polymerization.

### 3.1 Vegetable Oil Functionalization

Natural VOs may already contain triglycerides whose fatty acid chains possess reactive functional groups, such as double bonds, epoxy or hydroxyl groups, that can be directly used for polymerization. For instance, *a vegetable oil extracted from Vernonia galamensis (Cass.) Less*., a native Ethiopian plant, contains a high concentration of naturally epoxidized triglycerides (glyceryl trivernolate) in its seeds, which could be used as an alternative to conventional epoxy oils. However, such naturally occurring reactive moieties are rare among plant‐derived fatty acids. Consequently, various chemical modifications have been developed to introduce functional groups suitable for polymer synthesis.

Among the vegetable oils most frequently used in the studies reviewed and discussed in the following sections are castor oil (CO), soybean oil (SBO), and linseed oil (LO).

#### 3.1.1 Epoxidation

**SCHEME 1:**
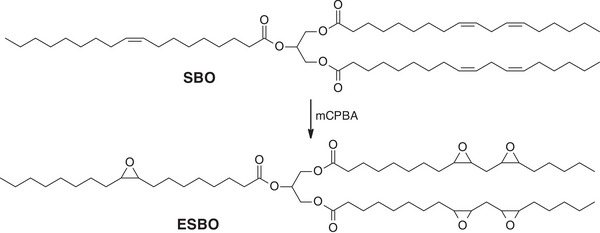
Production of epoxidized soybean oil (ESBO) from natural soybean oil (SBO) under the Prilezhaev conditions.

VOs are rich in unsaturated fatty acids, making them suitable precursors for the introduction of epoxide groups into their structure [14]. Industrially, epoxidized VOs are typically obtained thanks to the Prilezhaev reaction on unsaturated triglycerides [15, 16]. This process commonly uses peracids, such as *m*‐chloroperoxybenzoic acid (mCPBA), performic acid, or peracetic acid, for the epoxidation of alkene groups (Scheme 1). The reaction can be carried out using either preformed or in situ‐generated organic percarboxylic acid. However, handling peracids on a large industrial scale poses significant safety risks, that is why the in situ method is generally preferred. Moreover, epoxides are sensitive to acidic conditions, which can lead to the oxirane ring opening and the formation of multiple undesirable by‐products [17]. Thus, research on the synthesis of epoxidized VOs as safer and more effective ways is ongoing. As an example, a toxicity‐free epoxidation method was developed, using hydrogen peroxide, catalyzed by alumina [18]. Thanks to a 3‐axis experimental design on methyl ricinoleate, Parada Hernandez et al. were able to successfully and completely epoxidize castor oil in 6 h, compared to the 14–16 h required under Prilezhaev conditions. A selectivity of 89% was achieved, meaning that only a small proportion of alcohol groups were produced. Notably, this method offers a faster reaction time than conventional industrial processes while avoiding the use of hazardous and corrosive peracids.

#### 3.1.2 Hydroxylation

**SCHEME 2:**
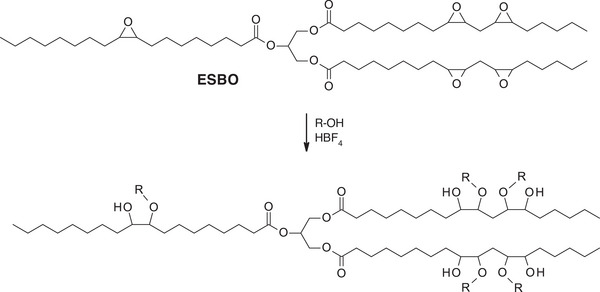
Hydroxylation of epoxidized soybean oil (ESBO) (R = ─CH_3_, ─CH_2_CH_2_OH, ─CH_2_CHOHCH_3_).

Some oils, such as castor oil, naturally contain hydroxyl groups in their structures, specifically ricinoleic acid, which has a hydroxyl group at the 12th carbon. However, most commonly used vegetable oils, such as soybean, sunflower, or linseed oils, lack hydroxyl functionalities along their fatty acid chains. The absence of these reactive groups limits their direct reactivity in certain polymerization processes. Therefore, the chemical introduction of hydroxyl groups through hydroxylation reactions becomes a crucial step to enhance their functionality and broaden their application. To introduce such groups, unsaturated VOs can be functionalized through the hydroxylation of epoxidized oils [19]. Usually, the oxirane groups are opened by reacting with ethylene or diethylene glycol, and using acids as catalysts, such as sulfuric, phosphoric, or *p*‐toluenesulfonic acids. They can also be hydroxylated thanks to ring‐opening reaction with methanol, 1,2‐ethanediol, or 1,2‐propanediol, and fluoroboric acid as a catalyst (Scheme 2) [20].

#### 3.1.3 Cyclic Carbonates from Epoxidized VOs

**SCHEME 3:**
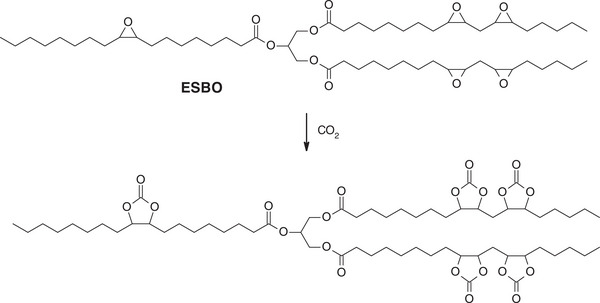
Synthesis of cyclic carbonate VOs from epoxidized VOs.

The functionalization of epoxidized vegetable oils to cyclic carbonates also represents a key strategy to enhance their reactivity toward the synthesis of non‐isocyanate polyurethanes (NIPUs). While the epoxidation of unsaturated fatty acid chains introduces reactive epoxy groups, these moieties alone are not directly suitable for the formation of the polymer. Converting epoxides into five‐membered cyclic carbonates provides a route to incorporate reactive functionalities into the oil structure. These cyclic carbonates serve as key intermediates and can undergo polyaddition reactions. An example of such reactivity will be discussed in Section 3.3.1. Cyclic carbonates can be synthesized from epoxidized molecules through a cycloaddition reaction with carbon dioxide (CO_2_) (Scheme 3). Various catalytic systems have been investigated to promote the formation of cyclic carbonates from epoxides, including calcium‐based catalysts or quaternary ammonium salts, both of which have shown promising activity and selectivity [21]. The incorporation of CO_2_, whether directly from gas or captured from industrial emissions, not only contributes to carbon valorization but also enhances the overall sustainability of the polymer synthesis process by reducing reliance on fossil‐derived reagents and lowering the carbon footprint.

### 3.2 Vegetable Oil‐Based Polyesters

#### 3.2.1 Polyester Synthesis

**SCHEME 4:**
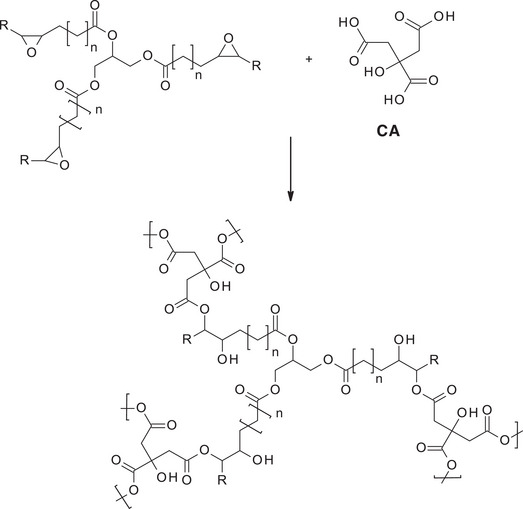
Reaction of epoxidized VOs with citric acid to obtain polyesters.

Epoxides are highly reactive electrophilic species that readily undergo ring‐opening reactions in the presence of acids, particularly Brønsted acids, which protonate the oxirane oxygen and facilitate nucleophilic attack. This reactivity has been widely exploited in the synthesis of polyesters from VO‐derived epoxidized monomers. In this context, multifunctional carboxylic acids, such as citric acid (CA), act as both crosslinkers and chain extenders by reacting with epoxide groups to form β‐hydroxy esters through esterification (Scheme 4) [22]. The reaction typically proceeds under mild conditions and allows the formation of highly branched or networked polyester structures, depending on the functionality of the acid and the epoxide precursor.

**SCHEME 5:**
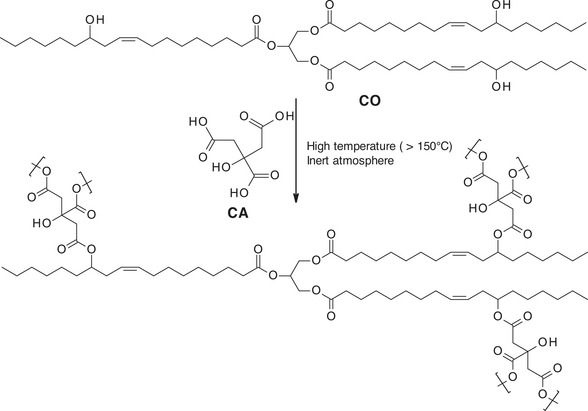
Esterification of ricinolein in CO with citric acid (CA).

Moreover, polyesters can also be prepared by the esterification at high temperature of ricinolein, the main constituent of CO, which contains a hydroxyl group, with multi‐acids such as citric or sebacic acids (Scheme 5) [23, 24]. Sometimes, a multi‐hydroxyl containing component, such as mannitol, is added as a chain extender [24], which can influence the polymer properties.

#### 3.2.2 Polyesters for Drug Delivery Systems

**FIGURE 3:**
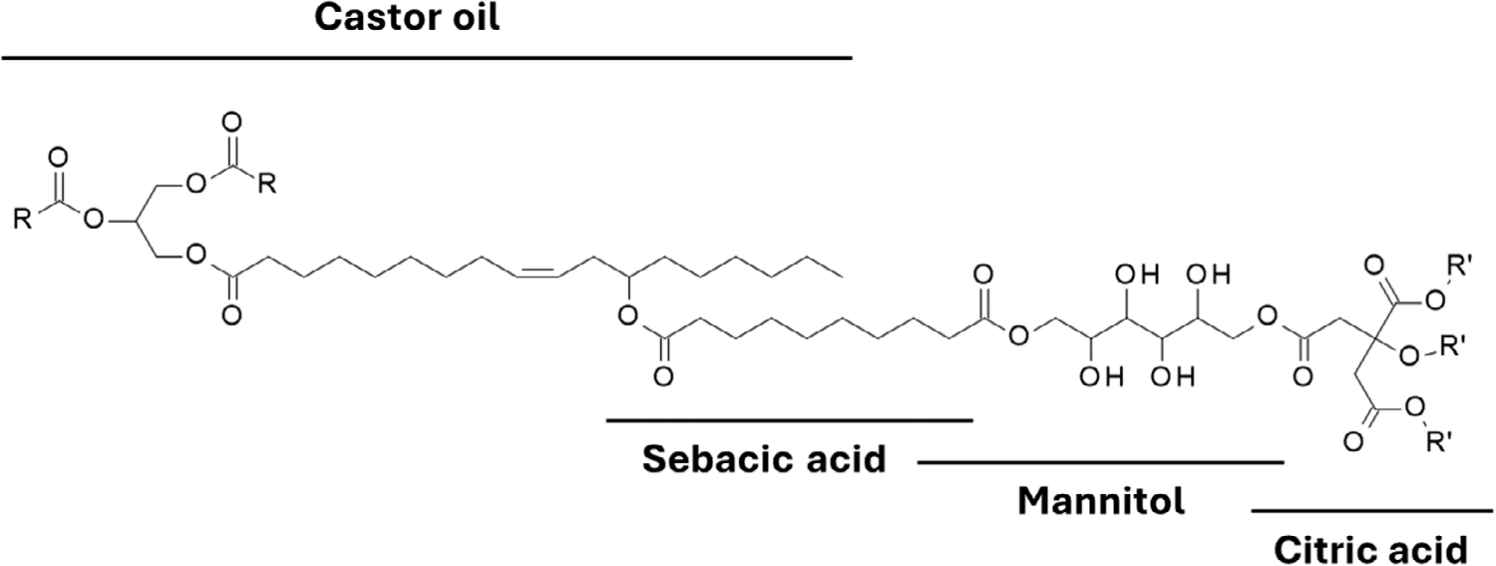
Castor oil‐sebacic acid‐mannitol‐citric acid polyester.

Castor oil‐sebacic acid‐mannitol‐citric acid polyester films (CO‐p(MCS)) have been developed as drug delivery systems [23, 24]. They were prepared via polycondensation between castor oil and a mixture of sebacic and citric acids, and D‐mannitol was added as a multifunctional hydroxylated crosslinker (Figure 3).

**FIGURE 4:**
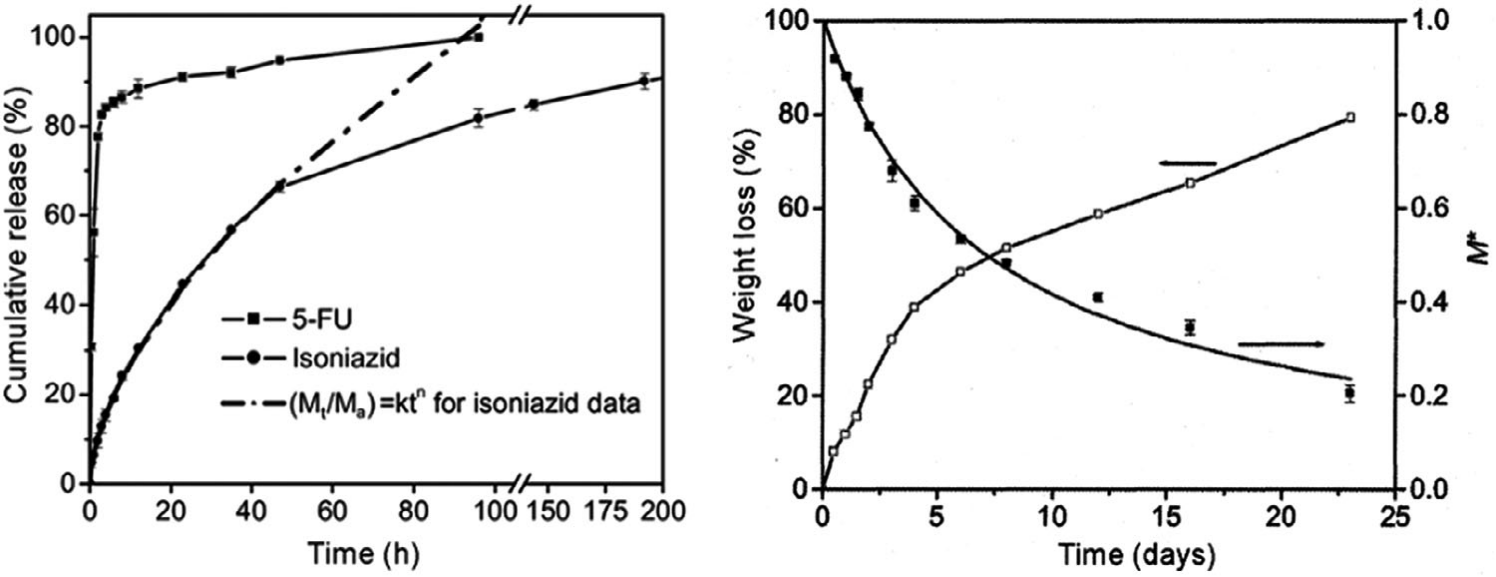
Left: Cumulative release of 5‐FU and isoniazid from CO‐p(MCS) polymer films; Right: degradation profile (M^*^ = M/M_0_, M^*^ = 1 at t = 0 days). Reproduced with permission [24]. Copyright 2012, Indian Academy of Sciences.

Hydrophilic drugs, 5‐fluorouracil (5‐FU) and isoniazid, used for anticancer and antituberculosis treatments, respectively, were loaded by solubilization into the monomer mixture prior to polymerization, and their release kinetics were studied under physiological simulated conditions (37°C, pH 7.4) (Figure 4). The polymer exhibited an initial burst release of 5‐FU, attributed to drug molecules adsorbed or weakly bound to the polymer surface (Figure 4A). Total cumulative release of 5‐FU was achieved in 48 h. In contrast, isoniazid showed a more sustained release profile, with complete release occurring over 16 days and no initial burst, highlighting the importance of drug–polymer interactions in modulating release behavior. Biocompatibility of the CO‐p(MCS) polymer was tested using human foreskin fibroblasts (HFF) cells, which adhered well to the polymer surface and reached full confluence (the cells occupied the full area of the polymer film) after 7 days, meaning excellent biocompatibility. Degradation studies under in vitro conditions reached 80% of mass loss after 23 days of study (Figure 4B). These results demonstrated that CO‐p(MCS) films possess promising biocompatibility and biodegradability for biomedical applications; however, further optimization is required to control drug encapsulation efficiency and minimize burst release.

Drug delivery systems for the sustained release of the anesthetic drug bupivacaine were developed using liquid polyesters, synthesized from CO and D,L‐lactic acid (LA) [25]. Initially, the authors designed a ricinoleic acid‐sebacic acid polyanhydride‐based formulation. However, the fast hydrolysis of the anhydride bonds under physiological conditions led to a burst release of the compound [26]. Subsequently, they developed a polyester where the presence of more hydrolytically stable ester bonds allowed a slower degradation rate and prolonged the release of bupivacaine. The polyester was synthesized with low molecular weight entities, which resulted in a low viscosity polymer and maintained a liquid state at 37°C (Table 3), making it injectable through a 22G needle for in vivo evaluation. Moreover, bupivacaine as a free base (log P of 3.31) was easily solubilized in these polymers, with drug loading capacities reaching up to 20% (w/w).

**TABLE 3:** Molecular weights Mn and Mw and viscosities of lactic acid/castor oil polymers.

LA/CO ratios	Mn/Mw	Viscosity at room temperature (cP)	Viscosity at 37°C (cP)
40/60	2800/2100	3400	900
30/70	2900/2100	1700	500

Hydrolytic degradation studies showed an initial 20% weight loss during the first week of study, after which the polymer weight remained relatively constant in the following weeks [25]. Thus, this slower degradation profile allowed a prolonged in vivo sensory anesthesia of 48 h, compared to 30 h observed with the poly(sebacic‐co‐ricinoleic acid) polymers, and enabled 24 h of motor block in rats.

**FIGURE 5:**
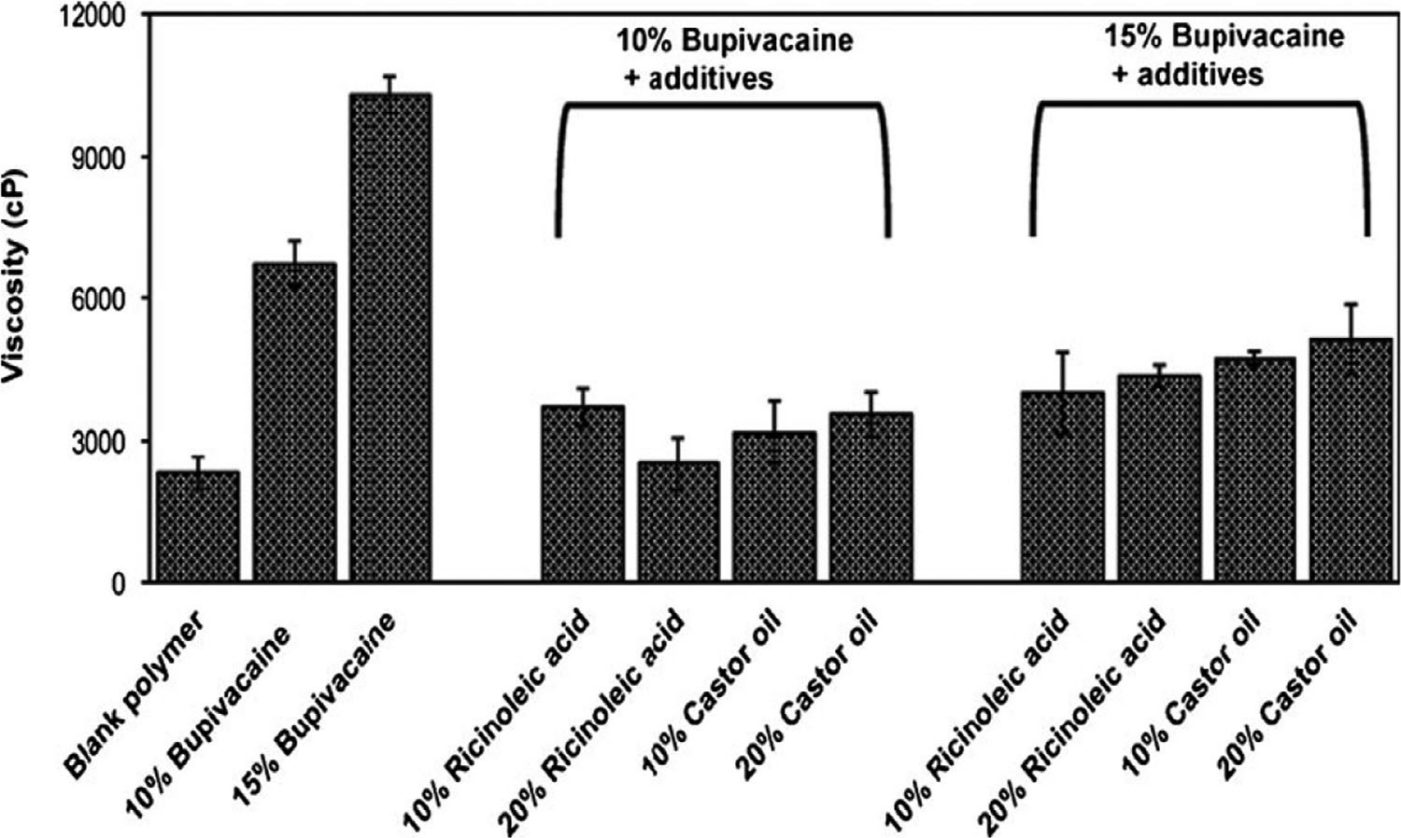
Effect on the viscosity of poly(DLLA:CO) (3:7) polymers containing bupivacaine (10 or 15% w/w) by the incorporation of additives (Ricinoleic acid or CO) in the polymer formulation. Reproduced with permission [28]. Copyright 2011, Springer Nature.

In vitro release studies showed that all polymer formulations released 60% of the solubilized molecule within 7 days. Formulations with 30% of LA showed burst releases of the molecule with up to 50 % of release in the first seven hours, no matter the encapsulation rate (7.5 or 10% w/w); whereas 40% of LA led to a linear release profile [27]. The increase of drug loading to 15% in the 30/70 LA/CO ratio polymers eliminated the burst effect, with only 10% of bupivacaine released in 6 h, enabling a prolonged sensory block lasting up to 96 h, and a motor block of 60 h [27]. This phenomenon is attributed to stronger polymer‐drug interactions facilitated by the hydrophobic character of the polymer, which contains 70% of CO. The concentration of bupivacaine could not be further increased, as a formulation of 20% w/w was too viscous for injection at the sciatic nerve site. Thus, the same group worked on the incorporation of low‐viscosity hydrophobic additives in order to decrease the room temperature viscosity of such polymers, aiming to maintain injectability while achieving a prolonged analgesic effect [28] (Figure 5).

The addition of ricinoleic acid (RA) or CO significantly reduced the viscosity of the formulations, from 6 700 cP (10% bupivacaine) or 10 300 cP (15% bupivacaine), down to approximatively 4 000 cP for both formulations. No significant difference was observed between RA and CO in terms of viscosity reduction. The additives also influenced the release rate of the molecule: formulations with CO achieved 85% cumulative release after 7 days, compared to 60% without the additive, and no burst effect was observed. It also affected the analgesic effect by prolonging the sensory block while shortening the motor block. Overall, the addition of CO into the poly(DLLA:CO) polymer lowered viscosity, facilitating the injection, and improved the in vivo efficacy results.

#### 3.2.3 Polyesters for Wound Dressings

**FIGURE 6:**
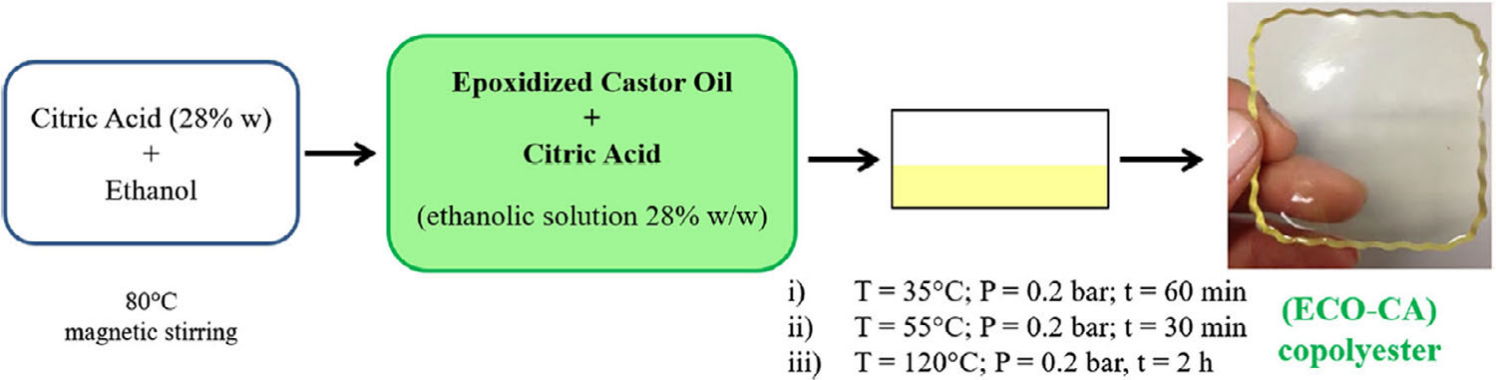
Synthesis of the (ECO‐CA) polyester films. Reproduced with permission. [22] Copyright 2019, Springer Nature.

In addition to their use in drug delivery, oil‐based polyesters have also been investigated for wound dressing applications. In this context, Parada Hernandez et al. reported the formation of polyester films, synthesized from epoxidized castor oil (ECO) and citric acid (CA), using an ethanol evaporation process under vacuum (Figure 6) [22].

Thanks to the non‐toxic epoxidation process detailed above (see paragraph 3.1.1) and a green copolymerization route, the ECO‐CA polymer film showed no cytotoxicity toward fibroblast cells, and additionally supported cell adhesion and growth on its surface. However, the mechanical properties and water uptake behaviors of the films still need to be thoroughly evaluated to determine their suitability for wound dressing applications.

**SCHEME 6:**
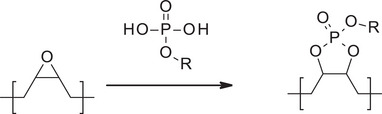
Reaction of phosphoester with epoxides to form cyclic phosphoesters.

Another approach to obtain biodegradable and biocompatible polyesters involves the use of phosphoester cross‐linked VO polymers. A process using CO was reported, in which the free hydroxyl groups of CO were phosphorylated using POCl_3_, yielding phosphorylated castor oil (PCO). This latter was then polymerized in a Teflon mold with either ESBO or epoxidized linseed oil (ELO) at 37°C, without any solvent or additive [29]. In this system, the phosphoester groups on PCO react with the epoxide functionalities of ESBO or ELO to form cyclic phosphoester linkages (Scheme 6) [30].

Hydrolysis experiments of the polymers were performed as phosphoester bonds are susceptible to hydrolytic degradation. The PSO samples (materials made from the polymerization of PCO and ESBO) containing a higher ratio of phosphorylated compound compared to the epoxides were rapidly hydrolyzed (in less than 4 days at 37°C in a buffer solution (0.01 m, Na_2_HPO_4_/NaH_2_PO_4_) (Table 4). This accelerated degradation was attributed to the acid‐catalyzed hydrolysis due to the excess of P‐OH groups in the polymer network. When the ratio reached stoichiometric balance (1:1) between phosphoester and epoxide groups, the hydrolysis duration increased to 21 days. The PLO samples (derived from PCO and ELO) showed a similar trend in hydrolysis behavior. However, since PLO contains more oxirane rings than PSO, the resulting polymer exhibited enhanced network density, leading to slower degradation.

**TABLE 4:** PCO, ESBO, and ELO ratios for polyphosphoester polymers and their duration for complete hydrolysis.

Sample	PCO (%)	ESBO (%)	ELO (%)	Complete hydrolysis (days)
PSO‐1	66	37	0	1
PSO‐2	60	40	0	4
PSO‐3	50	50	0	21
PSO‐4	40	60	0	21
PSO‐5	37	66	0	21
PLO‐1	66	0	37	4
PLO‐2	60	0	40	21
PLO‐3	50	0	50	27
PLO‐4	40	0	60	27
PLO‐5	37	0	66	27

In vivo experiments were done on PLO‐3 via subcutaneous implantation in rats. No inflammation on the implantation site was observed after two weeks, nor after 2 months, showing the biocompatibility of such polymers. Moreover, the polymer underwent complete degradation after 3 months in vivo. These results demonstrate that phosphorylated CO‐based polymers are both biocompatible and biodegradable, which are outstanding properties for biomedical applications.

### 3.3 Vegetable Oil‐Based Polyurethanes

#### 3.3.1 Polyurethane Synthesis

**SCHEME 7:**
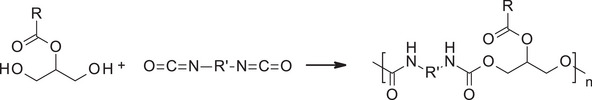
Synthesis of VO‐based polyurethane from a monoglyceride and a diisocyanate.

Polyurethanes are widely used in biomedical applications due to their biocompatibility and mechanical properties [31, 32]. Traditionally, they are produced by the reaction of polyols (called hydroxyl‐containing component (HCC)) with diisocyanates such as hexamethylene diisocyanate (HMDI), methylene diphenyl diisocyanate (MDI), toluene diisocyanate (TDI), or isophorone diisocyanate (IPDI) [33]. To enhance sustainability and introduce novel functionalities, this process has been adapted to incorporate triglycerides and their derivatives as renewable polyol sources, which can react with polyisocyanates to yield new materials with properties suitable for multiple biomedical applications (Scheme 7).

**SCHEME 8:**
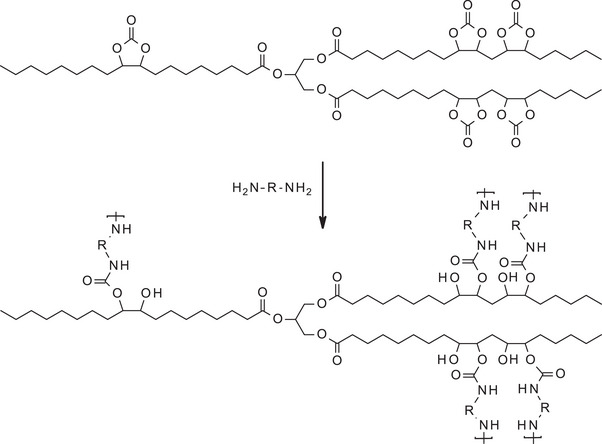
Production of non‐isocyanate polyurethanes (NIPUs) from functionalized soybean oil containing cyclic carbonates.

It should be noted that, non‐isocyanate polyurethanes (NIPUs) were recently developed as a safer and more sustainable way to synthesize polyurethanes [34]. Indeed, diisocyanates are produced by the reaction of amines with carbonyl dichloride (phosgene), which is a highly toxic and harmful component [35]. As previously discussed (see Section 3.1.3), cyclic carbonates can be synthesized from epoxidized vegetable oils through carbonation reactions, offering a promising route to replace toxic isocyanates. These cyclic carbonates serve as key intermediates in the production of NIPUs via polyaddition reactions with amines (Scheme 8). In this approach, the ring‐opening of the cyclic carbonate by a primary diamine leads to the formation of β‐hydroxyurethane linkages, without the release of harmful by‐products. The use of such renewable, bio‐based precursors not only enhances the environmental profile of the resulting polyurethanes but also imparts favorable properties such as improved biocompatibility, reduced toxicity, and potential biodegradability. This is particularly advantageous for biomedical applications [36], where the absence of residual isocyanates is crucial. Thus, NIPU synthesis from VO‐derived cyclic carbonates represents a sustainable and safer alternative to conventional polyurethane production.

#### 3.3.2 Polyurethanes for Drug Delivery Systems

**SCHEME 9:**
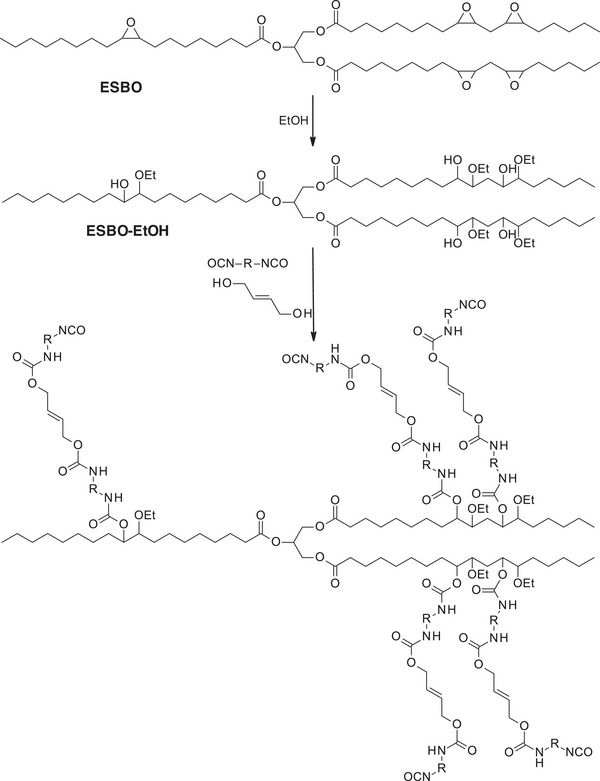
Synthesis of isocyanate‐terminated soybean oil derivative, synthesized with a diisocyanate (OCN‐R‐NCO) and hydroxy‐terminated polybutadiene, for the production of PU with LA‐EG chain extender.

SBO‐based polymer films intended for drug delivery applications were produced by the hydrolyzation of epoxidized soybean oil (ESBO) with ethanol, followed by polymerization with IPDI and hydroxyl‐terminated polybutadiene (HTPB), and an ethylene glycol‐lactic acid‐based chain extender (LA‐EG) (Scheme 9) [37]. The model drug, gabapentin, an anticonvulsant drug, was dissolved in DMSO and added to the reaction mixture before polymerization. The films were then formed using the solvent evaporation method.

**FIGURE 7:**
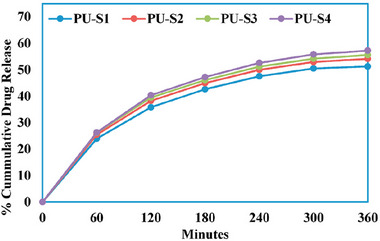
Cumulative drug release of gabapentin from EBO:HTPB polymers. Reproduced with permission [37]. Copyright 2024, Elsevier.

Four PUs were synthesized by varying the ratio of hydrolyzed ESBO (ESBO‐EtOH) and HTPB (Table 5) and exhibited different behaviors. An increased proportion of HTPB led to enhanced hydrophobicity of the polymer, as evidenced by higher contact angle values and reduced water absorption, due to the high lipophilicity of the material. Gabapentin release studies revealed an initial burst effect during the first hour, with approximately 25% of the drug released (Figure 7), likely due to the desorption of surface‐associated molecules. This was followed by a progressive and incomplete release phase, reaching around 50% of the drug released at 6 h. All four tested polymers showed a similar release profile, highlighting that the ESBO‐EtOH:HTPB ratio had no significant impact on the drug release kinetics.

**TABLE 5:** ESBO‐EtOH and HTPB ratios in PUs synthesis (all PUs were synthesized with 2.1 equivalents of IPDI and 1 equivalent of LA‐EG), and their contact angle and water absorption on the first day of study. Adapted from A. Iqbal et al. [37].

Sample	ESBO‐EtOH (%)	HTPB (%)	Contact angle (°)	Water absorption (%)
PU‐1	90	10	74.89	5.12
PU‐2	80	20	81.36	4.96
PU‐3	70	30	89.39	4.73
PU‐4	60	40	96.46	4.50

The same authors also developed an ESBO‐based polyurethane by the polymerization of a blend of hydrolyzed ESBO and PEG (400) instead of HTPB, with IPDI and the same LA‐EG chain extender [38].

Again, four PUs were synthesized by varying the ratio of ESBO‐EtOH and PEG (Table 6). A slight decrease in contact angle was observed when the ratio of PEG increased, suggesting enhanced surface hydrophilicity due to the incorporation of PEG. Water absorption remained relatively stable, with a modest increase corresponding to higher PEG proportions, as expected. However, the lack of available standard deviation data for these measurements limits the ability to assess the statistical significance of the observed differences.

**TABLE 6:** ESBO‐EtOH and PEG ratios in PUs synthesis (all PUs were synthesized with 2.1 equivalents of IPDI and 1 equivalent of LA‐EG), and their contact angle and water absorption on the first day of study. Adapted from A. Iqbal et al. [38].

Sample	ESBO‐EtOH (%)	PEG (%)	Contact angle (°)	Water absorption (%)
PU‐5	90	10	70.41	4.9
PU‐6	80	20	70.11	5.5
PU‐7	70	30	66.88	6.3
PU‐8	60	40	63.45	6.7

All PUs showed high gabapentin encapsulation efficiency, along with an initial burst release in the first hour (from 12.5% to 42.3% depending on the PU). The cumulative release reached up to 53% after 6 h (Table 7). In contrast to the previous study with ESBO:HTPB, a decrease in release was observed with increasing PEG content. Notably, no drug release was detected even after 6 h for PU‐8. This phenomenon was attributed to the increased swelling capacity of the polymer matrix, resulting in a slower release rate and an extended diffusion length [39, 40].

**TABLE 7:** Encapsulation efficiency and gabapentin release of ESBO: PEG‐based PU films. Adapted from A. Iqbal et al. [38].

Sample	Encapsulation efficiency	Drug released – 1 h (%)	Drug released – 6 h (%)
PU‐5	98.0	42.3	53.0
PU‐6	97.4	31.9	41.7
PU‐7	98.2	12.5	17.3
PU‐8	97.0	0	0

#### 3.3.3 Polyurethanes for Wound Dressings

Polyurethanes have also gained significant attention for wound dressing applications due to their favorable properties, such as biocompatibility, biodegradability, and their ability to maintain a moist environment that promotes faster healing [41]. In recent years, increasing interest has been directed toward the development of bio‐based polyurethanes derived from VOs. Several studies have reported the use of monoglyceride derivatives of VOs, which contain reactive hydroxyl groups and have been employed as polyols in reactions with various diisocyanates to tailor the polymer network. These VO‐based polyurethanes have been evaluated for their mechanical performance and cytocompatibility. Further efforts have been made to optimize their molecular structure or incorporate bactericidal agents into the formulation, with the goal of enhancing their functionality and therapeutic efficacy.

**FIGURE 8:**
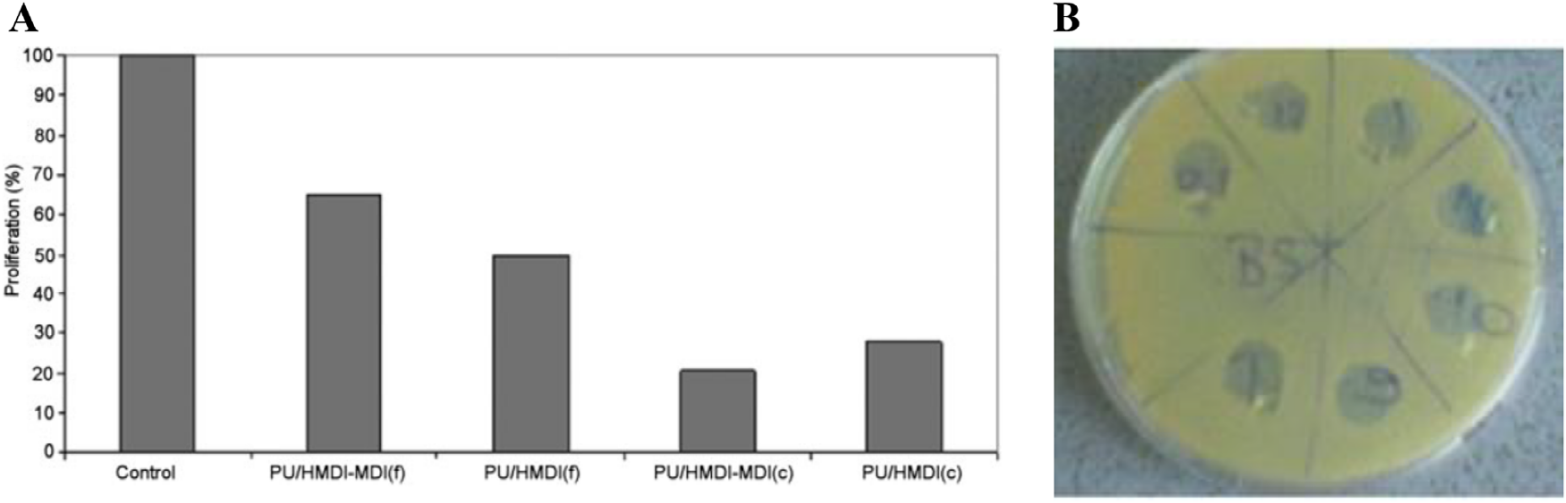
(A) Cell proliferation on the surface of the PUs. (B) Plate for the determination of antibacterial activity of the films against Bacillus subtilis using the agar diffusion method, shown after the removal of the film samples. Reproduced with permission [42]. Copyright 2010, Wiley.

A first study reported the synthesis of polyurethane‐based materials via the polymerization of linseed oil with MDI and/or HMDI, in the presence of Biocera A (bioceramic particles containing silver, known to have a bactericidal effect) [42]. The resulting polymers showed good mechanical properties and surface hydrophilicity, with contact angles below 80°. However, they did not show an acceptable cytocompatibility, with cell proliferation ranging from 20% to 70% (Figure 8A), which was attributed to residual metal catalysts entrapped during the process (cobalt octanoate and lead naphthenate). These catalysts were used as drying agents to facilitate the polymerization step, although their exact concentrations were not specified. These findings highlight the need to develop catalyst‐free or alternative synthetic routes to improve the cytocompatibility of the films. The antibacterial performance of the polymers was tested: while no inhibition zone was observed in standard diffusion tests, the absence of bacterial proliferation in areas previously occupied by the films suggests a potential surface‐associated antibacterial effect (Figure 8B).

Linseed oil‐based PUs were also synthesized using TDI and showed mechanical properties comparable to those obtained with MDI or HMDI [43]. All polymers were hydrophilic, with contact angles below 90°, and their flexibility depended on the rate of polymerization and cross‐linking, which were modulated through the use of catalysts (Table 8). The PUs are named according to the presence of catalysts or not: “NC” (no catalyst) and “WC” (with catalyst) for the polyurethane synthesis using 0.02% of calcium octoate, and “nc” (no catalyst) and “c” (with catalyst) for the cross‐linking stage using 0.6 wt.% of calcium octoate. Without catalysts, the degree of polymerization was lower, resulting in more flexible films, decreased contact angles, and increased water absorption. These characteristics contributed to improving mechanical and absorption properties, as well as enhanced cytocompatibility compared to catalyst‐containing formulations. As a result, catalyst‐free PUs appear to be more promising candidates for wound dressing applications.

**TABLE 8:** Mechanical and absorption properties of linseed oil‐based PUs. Adapted from G. Gultekin et al. [43].

Sample	Tensile strength (MPa)	Elongation at break (%)	Contact angles (°)	Water absorption (%)
PU‐NC‐c	5.4	25.2	86	0.33
PU‐NC‐nc	2.2	55.5	50	9.87
PU‐WC‐c	5.6	28.9	72	1.13
PU‐WC‐nc	2.2	64.1	62	9.08

Bactericidal agents have also been loaded into CO‐based PU films for wound dressing applications. In particular, glycidyltriethylammonium chloride (GTEAC), a quaternary ammonium compound with known antimicrobial properties, was integrated during PU synthesis. Films containing 50% w/w of GTEAC (PU‐50), showed antibacterial activity, with reductions of 99% and 100% against *E. coli* and *S. aureus*, respectively, without exhibiting any cytotoxic effects (Table 9) [44]. Moreover, water absorption and surface hydrophilicity of films, key elements of wound dressings, were improved due to the hygroscopic nature of the quaternary ammonium group of GTEAC, which promotes hydration of the film surface.

**TABLE 9:** CO‐based PU films with encapsulated GTEAC, exhibiting antibacterial activities. Adapted from A. Yari et al. [44].

Sample	GTEAC (%)	Tensile strength (MPa)	Elongation at break (%)	Contact angle (°)	Antibacterial reduction *E. coli* (%)	Antibacterial reduction *S. aureus* (%)
PU‐0	0	0.49 ± 0.02	26 ± 1	69 ± 4	2.2	3.5
PU‐30	30	0.48 ± 0.03	28.4 ± 0.9	50 ± 3	5.1	13.1
PU‐40	40	0.43 ± 0.07	31 ± 1	32 ± 2	9.3	21.8
PU‐50	50	0.40 ± 0.03	34 ± 2	28 ± 3	99.1	100.0

**SCHEME 10:**
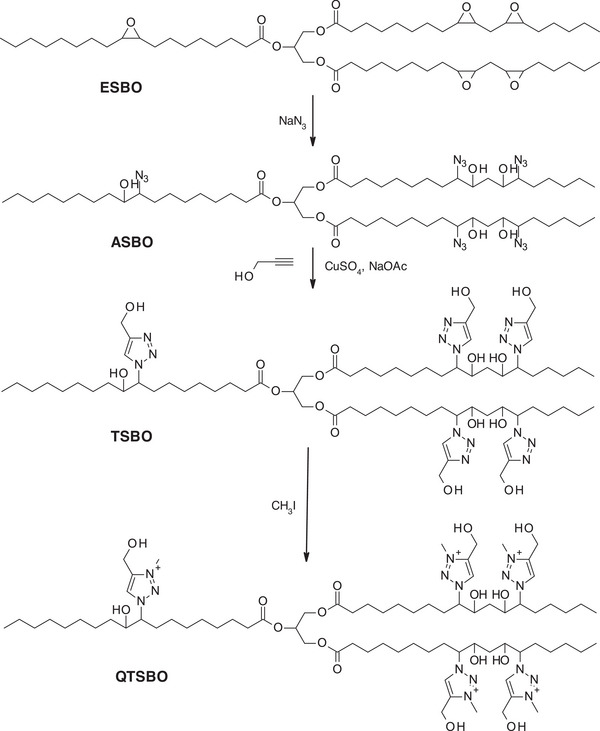
Synthesis pathway to synthesize QTSBO from ESBO.

As an alternative to loading external antibacterial agents into the polymer matrix, another strategy involves the direct incorporation of antibacterial functionalities into the polymer backbone. In this context, ESBO was chemically modified to introduce 1,2,3‐triazolium groups, known to have an antibacterial effect [45]. The modification process began with the synthesis of azide‐functionalized soybean oil (ASBO) via ring‐opening epoxide groups with sodium azide (NaN_3_) (Scheme 10). Then, triazole groups were formed by a click reaction with propargyl alcohol in the presence of CuSO_4_ and NaOAc. Finally, alkylation of the resulting 1,2,3‐triazole functional soybean oil (TSBO) with methyl iodide yielded 1,2,3‐triazolium‐functionalized soybean oil (QTSBO), which can be used as a reactive monomer in polyurethane synthesis, allowing for antibacterial activity to be embedded directly within the polymer structure [46].

QTSBO alone or in combination with CO was then used for polymerization with IPDI as diisocyanate, resulting in flexible PU materials. The incorporation of CO was found to be important for obtaining good mechanical properties, notably by increasing the tensile strength of the resulting films. In contrast, films made only from QTSBO were brittle (Table 10) and therefore unsuitable for wound dressing applications. All synthesized polymers exhibited excellent cytocompatibility (cell viability > 89%). Moreover, polymers containing 1,2,3‐triazolium groups showed 100% bacterial reduction against multiple microbial strains (*P. aerugiosa, S. aureus, C. albicans*), whereas no antibacterial activity was observed in polymers lacking these cationic groups. Thus, these new types of VO‐based PUs with additional cationic triazolium groups would be suitable for wound dressing applications with additional antibacterial properties.

**TABLE 10:** Mechanical properties of VO‐based PUs made of QTSBO/CO. Adapted from H. Gholami et al. [45].

Samples	QTSBO (%)	CO (%)	Tensile strength (MPa) (wet)	Elongations at break (%) (wet)	Contact angles (°)
PU‐1	0	100	0.11 ± 0.03	149.3 ± 7.8	85 ± 3
PU‐2	50	50	5.48 ± 0.31	132.9 ± 5.3	71 ± 6
PU‐3	70	30	10.10 ± 0.41	216.6 ± 8.1	57 ± 4
PU‐4	100	0	ND (brittle)	ND (brittle)	ND

#### 3.3.4 Non Isocyanate Polyurethanes for Wound Dressings

In addition to conventional PU formulations, ammonium‐functionalized PU has also been synthesized through isocyanate‐free routes, expanding the potential for safer and more sustainable biomaterials. For example, VO‐based NIPUs were produced by reacting carbonated soybean oil (CSBO) with diamines: 1,4‐butanediamine (BDA, also known as putrescine) and cyclohexanobis(methylamine) (CHM) at different ratios [47]. A high proportion of CHM resulted in brittle polymer films, while lower CHM content improved mechanical properties, which is essential for wound dressing applications. Notably, a 50% CHM ratio enhanced the elongation at break by nearly 200% (Table 11), demonstrating that this formulation yields more flexible and mechanically suitable VO‐based NIPU films for biomedical use.

**TABLE 11:** Elongation at break of CSBO‐based NIPUs synthesized with different ratios of BDA and CHM. Adapted from M. Morales‐ González et al. [47].

Samples	BDA (% molar)	CHM (% molar)	Elongation at break (%)
NIPU‐1	100	0	81.13 ± 18.82
NIPU‐2	75	25	145.89 ± 39.69
NIPU‐3	67	33	207.65 ± 31.05
NIPU‐4	50	50	273.21 ± 17.62

**SCHEME 11:**
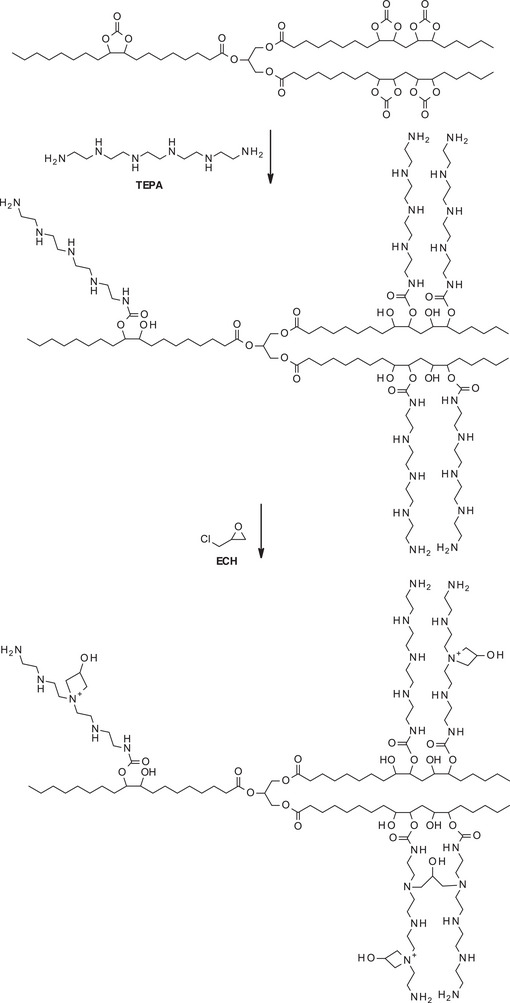
Synthesis of soybean oil‐based PU, modified by ECH.

NIPUs from CSBO were also produced using tetraethylenepentamine (TEPA) as the diamine component [48]. To introduce antibacterial functionality, the available, the available secondary amines of TEPA were reacted with epichlorohydrin (ECH) to form azetidinium groups (Scheme 11), being known to have an antibacterial activity [49].

These CSBO‐based NIPUs showed antibacterial activities against *E. coli* and *S. aureus*, with bacterial growth reductions of 100 and 98.27%, respectively (Table 12). Interestingly, the NIPU lacking azetidinium groups (NIPU‐T) also showed high bacterial efficacy against both bacterial strains. Surface charge analysis of the polymers was investigated and revealed that NIPU‐T possessed a significant positive zeta potential (ξ = +34.82 mV), attributed to the protonation of the secondary amines into dialkylammonium hydroxide species (R‐NH_3_^+^ OH^−^) at physiological pH. As expected, the azetidinium‐functionalized NIPU (NIPU‐Az) had an even higher positive charge (ξ = +43.55 mV). These findings suggest that the positive surface charge of the polymers plays a key role in their antibacterial performance, regardless of the specific functional group involved.

**TABLE 12:** ξ potential and bacterial reduction of NIPUs. Adapted from H. Gholami et al. [48].

Samples	ξ (mV)	*E. Coli*	*S. Aureus*
NIPU‐T	+34.82 ± 2.01	99.63 ± 0.18	98.09 ± 0.28
NIPU‐Az	+43.55 ± 1.98	100 ± 0	98.27 ± 0.41

### 3.4 Hybrid Organic/Inorganic Materials

#### 3.4.1 Hybrid Materials Synthesis

VOs have also been explored as renewable building blocks for the synthesis of hybrid organic–inorganic materials, offering a sustainable route to tailor‐made functional polymers. Two main classes of hybrid materials can be distinguished [50, 51].

**FIGURE 9:**
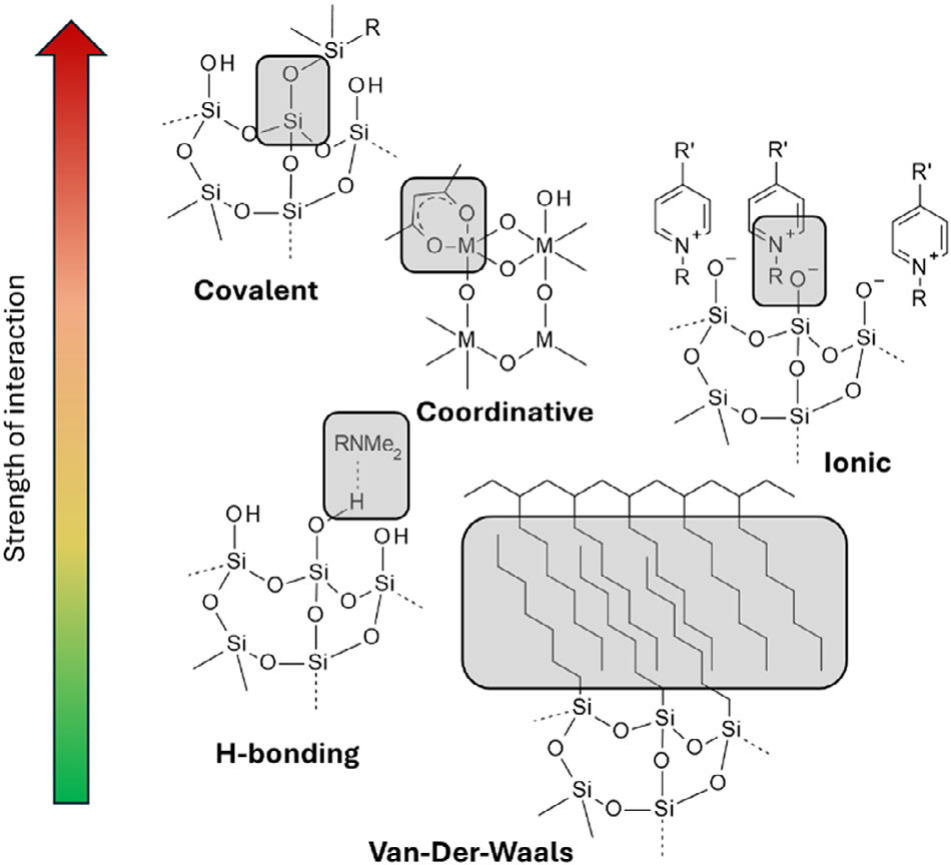
Interactions in hybrid polymers and their relative strength. Adapted from G. Kickelbick [51].

Class I hybrids are defined by weak interactions, such as H─bonding, Van Der Waals interactions, between the organic and inorganic components (Figure 9). They are typically obtained by the physical blending of the organic and the inorganic parts and are often referred as “composites”.

Class II hybrids, in contrast, involve strong chemical bonds, covalent, coordinative, or ionic, between the organic and inorganic moieties (Figure 9). In these systems, the organic phase forms a network of carbon atoms and heteroatoms, while the inorganic component is generally composed of a metal oxide framework (silicon, titanium, or zinc) [52]. The main advantage of these systems is that the resulting materials display intermediate properties between polymers and glasses, which can be finely tuned according to the ratio and nature of the components. These include mechanical strength, thermal resistance, optical properties, and chemical stability.

**FIGURE 10:**
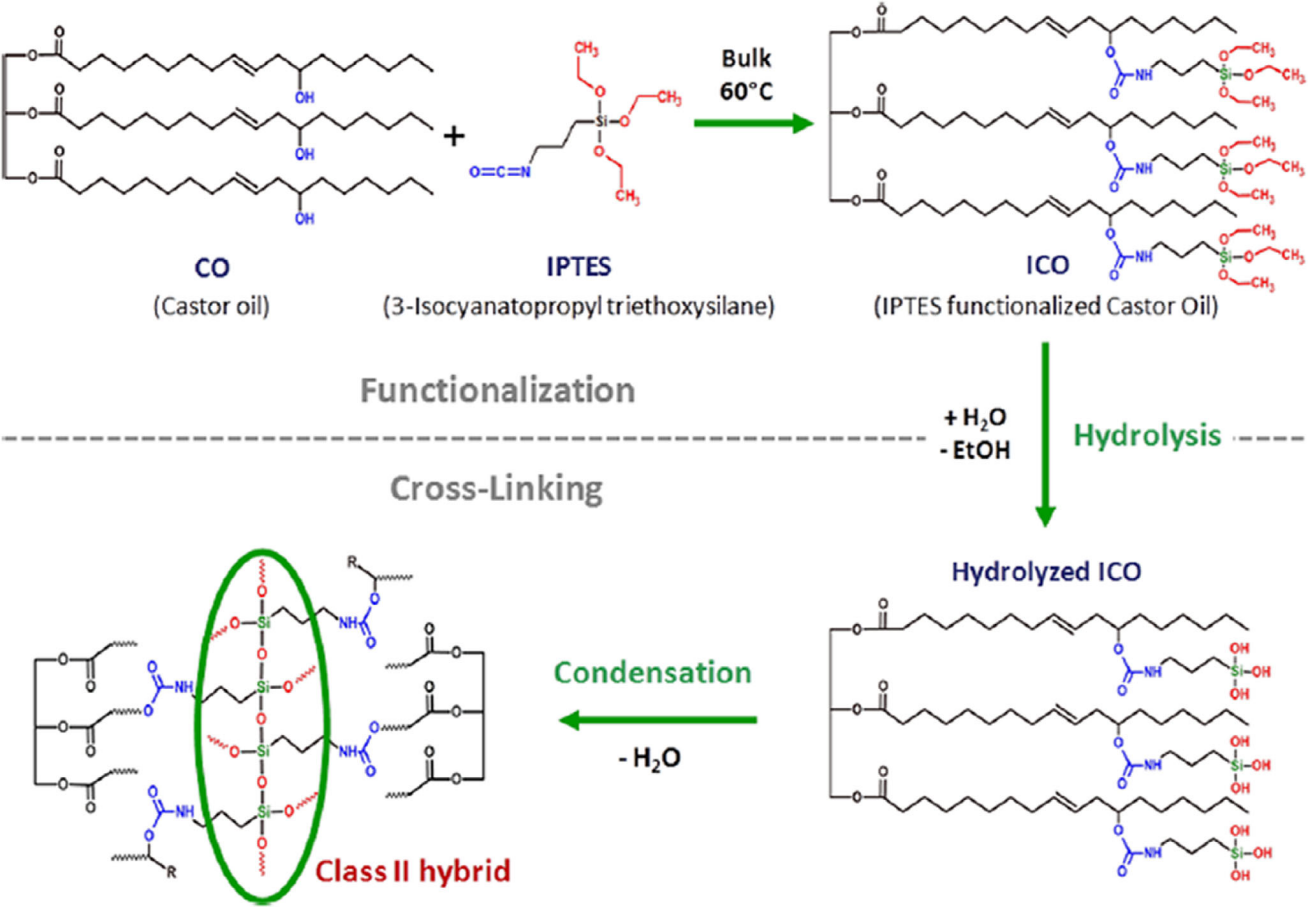
Synthesis and cross‐linking of IPTES functionalized CO. Reproduced with permission. [53]. Copyright 2017, American Chemical Society.

Thanks to advances in sol–gel chemistry, this approach has been adapted to VOs. In particular, CO, which naturally contains hydroxyl groups along its backbone, can be chemically functionalized using alkoxysilane‐containing isocyanates. A commonly used coupling agent is 3‐isocyanatopropyl triethoxysilane (IPTES), which can form urethane bonds with the free hydroxyl group of CO, allowing the production of polymers thanks to the hydrolysis and condensation of the added alkoxysilane groups (Figure 10). The degree of silylation can be controlled by adjusting the proportion of IPTES used in the synthesis, thereby modulating the final inorganic content of the hybrid polymer after cross‐linking and its mechanical properties. This ratio directly affects the reaction kinetics, which can be accelerated by the use of catalysts, and by adjusting the pH and temperature conditions. Such hybrid materials have been developed for healthcare applications, especially in drug delivery and in wound dressings.

#### 3.4.2 Hybrid Materials for Drug Delivery Systems

**FIGURE 11:**
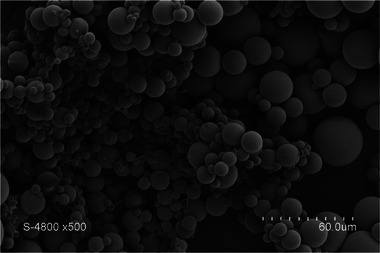
Solid hybrid microparticles obtained by the emulsification of ICO. Reproduced with permission [53]. Copyright 2017, American Chemical Society.

Microparticles were developed as drug delivery systems using sol–gel crosslinking of silylated castor oil (ICO) emulsified in an aqueous thermosensitive gel phase. The resulting spherical particles showed sizes around 100 µm depending on the emulsion process (Figure 11) (60°C; SnOct catalyst; pH 3; 8 days) [53].

These lipophilic particles were revealed to be highly effective drug delivery systems for lipophilic drugs, such as ibuprofen or estradiol [53, 54, 55]. The therapeutic molecules were encapsulated with a high loading efficiency of 95%, and their release was complete under in vitro conditions, within 10 h for ibuprofen and 7 days for estradiol. Moreover, no tissue inflammation was observed over a 28‐day period following subcutaneous injection in mice, demonstrating good biocompatibility.

**FIGURE 12:**
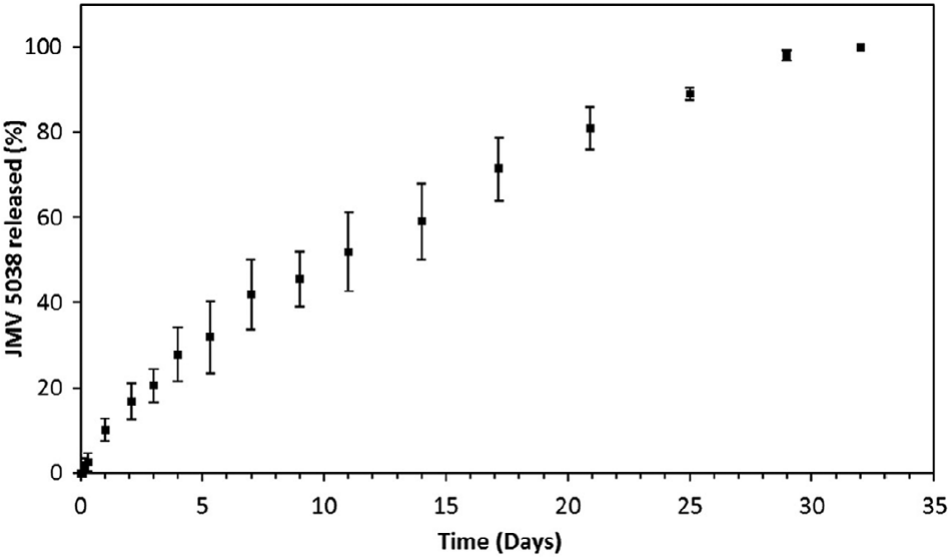
In vitro cumulative percentage of JMV5038 release from hybrid nanoparticles of ICO in a Phosphate‐Buffered Saline (PBS)/octanol biphasic system at 37°C. Reproduced with permission [59]. Copyright 2021, Elsevier.

**FIGURE 13:**
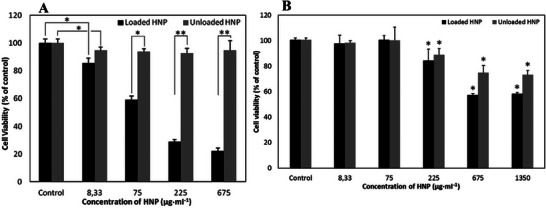
Cell viabilities of loaded (JMV5038) and unloaded hybrid particles of ICO. A) A375 melanoma cell line; B) NIH3T3 fibroblast cell line. Reproduced with permission [59]. Copyright 2021, Elsevier.

More recently, this type of functionalized CO‐based microparticles was used as a formulation system for JMV5038, a lipophilic cytotoxic drug highly effective against melanoma cell lines [56, 57, 58]. JMV5038 was revealed to be sensitive to acidic pH, thus the formulation was adapted by using a neutral medium, allowing successful encapsulation of the molecule at a 2% (w/w) rate (60°C; SnOct catalyst; pH 6; 10 days) [59]. Release kinetic studies showed a constant release over 30 days with no burst effect (Figure 12). Moreover, the released JMV5038 from these microparticles retained its anticancer activity against melanoma cells (Figure 13A). The particles proved to be cytocompatible to a certain extent (Figure 13B): a slight cytotoxicity of unloaded particles was observed at higher concentrations (> 225 µg.mL^−1^), attributed to residual tin‐based catalyst (SnOct) used in the sol–gel process. At lower concentrations, these particles represent a promising cutaneous drug delivery system for JMV5038 in melanoma treatment.

#### 3.4.3 Hybrid Materials for Wound Dressings

**FIGURE 14:**
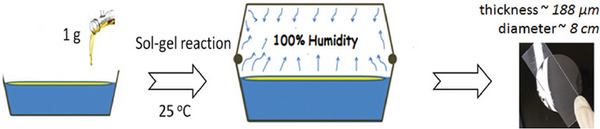
Production of VO‐based hybrid film in a solvent‐ and heat‐free method. Reproduced with permission [60]. Copyright 2017, Royal Society of Chemistry.

Following the same concept, hybrid films based on ICO were developed using a solvent‐ and heat‐free process (Figure 14) [60]. In this method, a thin layer of silylated CO was poured onto the surface of water, where sol–gel cross‐linking took place at the air–water interface, leading to the formation of thin hybrid films.

The mechanical properties of these films were assessed and showed a clear influence of the inorganic ratio on the Young's modulus (Table 13). Indeed, a higher ratio of inorganic matter (silylation rate = 80%) led to stiffer films (63 MPa), whereas films with a lower silylation rate (33%) were significantly more flexible, exhibiting a modulus of 4.3 MPa. Contact angle values were below 90° for all films, indicating a hydrophilic character of the film surface, attributed to the presence of siloxane moieties. These combined features, flexibility, and surface hydrophilicity, make these films adequate for wound dressing applications.

**TABLE 13:** Contact angles and Young's moduli of VO‐based films made of ICO of various silylation rates.

Samples	Silylation rates	Contact angles (°)	Young's moduli (MPa)
ICO‐F1	80%	85 ± 6	63
ICO‐F2	67%	80 ± 7	27
ICO‐F3	50%	78 ± 5	11
ICO‐F4	33%	72 ± 2	4.3

**FIGURE 15:**
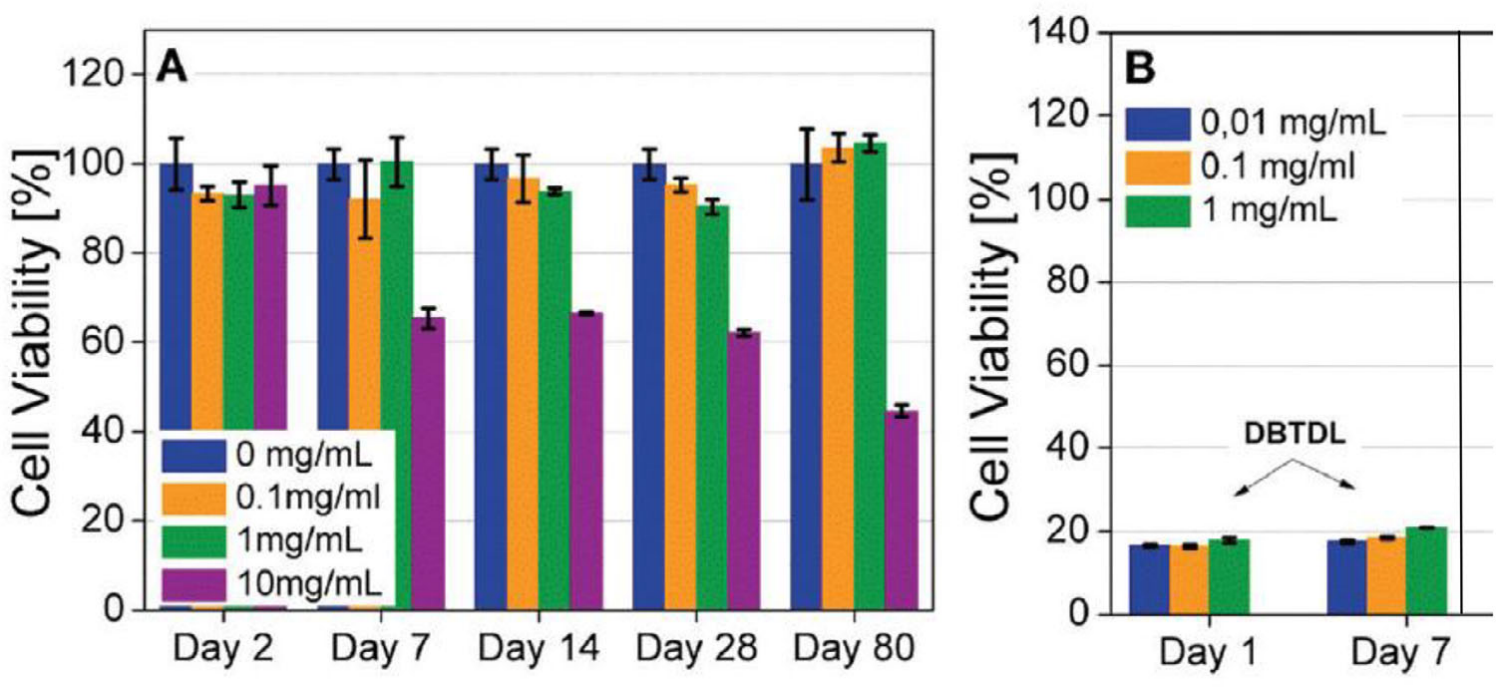
Cytotoxicity of (A) the hybrid film ICO‐F1; (B) the pure catalyst (DBTDL) on fibroblasts, expressed by a percentage of cell viability as a function of incubation time at different concentrations. Reproduced with permission [60]. Copyright 2017, Royal Society of Chemistry.

Moreover, the cytocompatibility of one of the films (ICO‐F1) was evaluated on fibroblast cells at different film concentrations (in mg.mL^−1^). Excellent cell viability (> 90%) was observed at concentrations of 0.1 and 1 mg.mL^−1^ over a two‐month testing period (Figure 15A). However, at a higher concentration (10 mg.mL^−1^), cell viability decreased to 67% after 7 days and further declined to 45% after 2 months. This decrease at high concentrations was attributed to the higher concentration of the tin‐based catalyst (dibutyltin dilaurate, DBTDL) used for the sol–gel cross‐linking. Cytotoxicity tests on pure DBTDL confirmed this hypothesis, showing less than 20% of cell viability at a concentration of 0.01 mg.mL^−1^ after 24 h (Figure 15B). To sum up, these VO‐based hybrid films showed favorable mechanical properties and cytocompatibility, particularly at moderate concentrations, making them good candidates for wound dressing applications.

**SCHEME 12:**
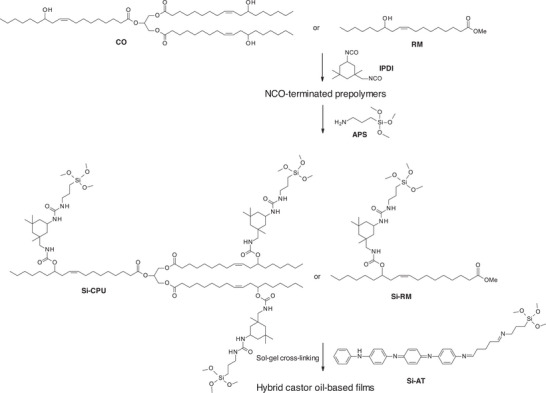
Preparation of hybrid castor oil‐based films from Si‐CPU, Si‐RM, and Si‐AT.

For such applications, new hybrid polyurethane/siloxane‐castor oil‐based membranes were developed [61]. NCO‐terminated urethane prepolymers were first prepared by the reaction of IPDI on castor oil and/or ricinoleic methyl ester (RM). These latter were then functionalized by (3‐aminopropyl)trimethoxysilane (APS) in the presence of DBTDL as a catalyst, yielding methoxysilane‐terminated urethane prepolymers, Si‐CPU from CO, and Si‐RM from RM (Scheme 12). Then, sol–gel cross‐linking was performed using different ratios of Si‐CPU and Si‐RM by a casting evaporation method (80°C for 12 h, then 100°C for 2 h). The residual DBTDL from the precursor synthesis acted as a catalyst for the sol–gel cross‐linking. Additionally, a trimethoxysilane‐terminated aniline tetramer (Si‐AT), known for its antimicrobial properties, was incorporated into the formulation as an active component [62, 63]. It is worth mentioning that the final dressing membranes were subjected to a disinfectant cleaning by immersion into ethanol (70% w/w) for 24 h followed by rinsing in distilled water for another 24 h before further in vitro tests.

The hybrid films were first evaluated without the addition of Si‐AT to determine the most suitable Si‐CPU/Si‐RM ratio for optimal film properties (Table 14). Films obtained from pure Si‐CPU were found to be hard and brittle. The addition of Si‐RM improved the flexibility of the resulting polymers, allowing the production of a completely flexible polymer, NEASiPU4 (Table 14). Consequently, this formulation was selected for the subsequent incorporation of Si‐AT. All films showed hydrophobic behavior, with contact angles exceeding 90° and water absorption rates around 2%, which is consistent with the hydrophobic natures of the carbon backbones of CO and RM, as well as the aromatic rings of AT. However, upon doping the hybrid films with camphorsulfonic acid (CSA) by immersion for 48 h in a CSA solution (2 mol.L^−1^), a significant decrease in contact angle values and an increase in water absorption were observed (d‐EASiPU1‐3, Table 14). This effect is attributed to the protonation of the amine groups in AT by CSA, which enhances their hydration. Thus, doping improved the hydrophilicity of the films, making them more suitable for wound dressing applications.

**TABLE 14:** Composition of hybrid films and their contact angles and water absorption values. Adapted from R. Gharibi et al. [61].

Samples	Si‐CPU (g)	Si‐RM (g)	Si‐AT (g)	Elongation at break (%)	Contact angles (°)	Water absorption (%)
NEASiPU1	3.33	0	0	Brittle	ND	ND
NEASiPU2	3.33	1.66	0	Brittle	ND	ND
NEASiPU3	3.33	2.50	0	Semiflexible	ND	ND
NEASiPU4	3.33	3.33	0	38.4 ± 2.2	95.2 ± 2.7	2.6 ± 0.2
EASiPU1	3.33	3.33	0.30	36.2 ± 0.9	95.3 ± 2.1	2.1 ± 0.1
EASiPU2	3.33	3.33	0.50	20.2 ± 0.5	97.1 ± 2.4	1.9 ± 0.2
EASiPU3	3.33	3.33	0.70	16.1 ± 0.7	106.5 ± 4.2	1.2 ± 0.1
d‐EASiPU1^a^	3.33	3.33	0.30	ND	84.5 ± 3.2	10.6 ± 0.5
d‐EASiPU2^a^	3.33	3.33	0.50	ND	57.6 ± 2.6	19.2 ± 0.8
d‐EASiPU3^a^	3.33	3.33	0.70	ND	48.3 ± 3.4	25.4 ± 1.1

^a^ Doping by immersion in a camphorsulfonic acid solution at 2 mol.L^−1.^

Moreover, their cytocompatibility was assessed on fibroblasts, showing 100% cell viability after 3 days. Interestingly, the doped membranes even showed an increased cell proliferation. Finally, thanks to the incorporation of AT, the doped films showed excellent antibacterial and antifungal activities, with reduction rates exceeding 87% against *S. Aureus*, *P. Aeruginosa*, and *C. Albicans* (Table 15). Notably, the formulation d‐EASiPU3, which contained the highest amount of AT, achieved complete microbial reduction across all three tested strains.

**TABLE 15:** Bacterial reduction percents of hybrid films. Adapted with permission [61]. Copyright 2015, American Chemical Society.

Samples	*S. aureus* (%)	*P. aeruginosa* (%)	*C. albicans* (%)
NESiPU4	0	0	0
d‐EASiPU1	87.2	89.3	88.7
d‐EASiPU2	99.1	98.5	98.3
d‐EASiPU3	100	100	100

**FIGURE 16:**
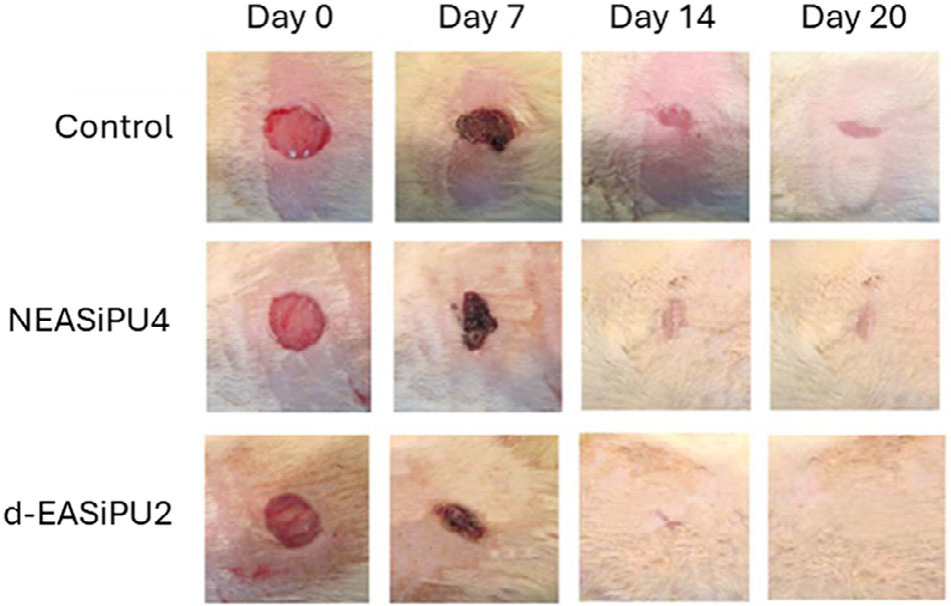
Photographs of the wound healing process in a rat model using NESiPU4 and EASiPU2 films, compared to a control group (without film). Reproduced with permission [61]. Copyright 2015, American Chemical Society.

In vivo wound healing studies were performed on a rat model to evaluate the benefits of using these hybrid films compared to a control without films [61]. The d‐EASiPU2 formulation was selected for this study, as it showed a good antibacterial activity and the highest proliferation rate. With this film, wounds showed 92% closure in 14 days, compared to 72% for NEASiPU4 (the same formulation without the antibacterial agent AT) and 63% for the control (Figure 16). After 20 days, complete wound closure was observed when d‐EASiPU2 was used, along with full tissue regeneration, including the development of dermal papillae and hair follicles. Thus, these hybrid films not only offer effective wound protection but also promote enhanced cell proliferation, adequate water uptake, and strong antimicrobial properties.

## 4 Unmodified Vegetable Oil‐Based Materials

### 4.1 Composites or Class I Hybrid

As defined in Section 3.4.1., composites of VOs are formed through the physical association of triglyceride‐based oils with other molecules, without the formation of chemical bonds. In contrast with the previously discussed chemically modified oils, this section will focus on unmodified VOs, used in combination with synthetic polymers. Most studies rely on an association with poly(lactic‐co‐glycolic acid) (PLGA), which is widely used in biomedical applications, due to its high biocompatibility and biodegradability. These physically blended systems will be discussed in the context of drug delivery applications or the development of wound dressing materials.

#### 4.1.1 Composites for Drug Delivery Systems

**FIGURE 17:**
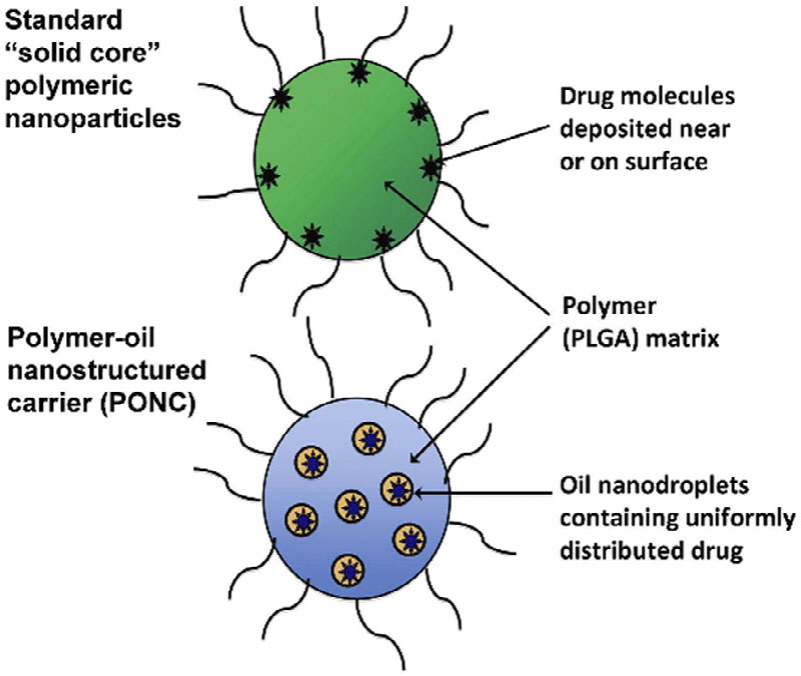
Comparison between two PLGA nanoparticle designs used as drug delivery systems: conventional polymeric nanoparticles (top), and polymer‐oil nanostructured carriers (PONC, bottom). Reproduced with permission [65]. Copyright 2014, Elsevier.

PLGA‐based polymers can easily be hybridized with oils to produce PLGA‐lipid composites, where the oil is embedded within the core of the system, which enhances their performance for drug delivery applications. Hence, a new class of hybrid nanocarriers, known as polymer‐oil nanostructured carriers (PONC), was designed to encapsulate highly lipophilic drugs (Figure 17) [64]. Such PONC combine the advantages of a lipid‐based carrier (efficient encapsulation of highly lipophilic drugs) with those of a polymeric carrier (e.g. uniform particle size, good stability). Moreover, they are known to have a reduced burst release effect and improved therapeutic action of the encapsulated drug, compared to conventional PLGA‐nanoparticles, thanks to the intracellular diffusion of drug molecules facilitated by the presence of oil.

Only a few examples of such systems were published, as most studies rely on commercially available modified VOs, such as caprylic and capric esters from coconut and palm kernel *oils* (e.g. Captex 200, propylene glycol dicaprylate/dicaprate, C8‐C10) [65, 66] or the surfactant PEG‐40 Hydrogenated Castor Oil (Acrysol K‐150) [67].

Nevertheless, composites using VOs have been developed for the delivery of indomethacin (IMC), a lipophilic non‐steroidal anti‐inflammatory agent (Log p = 3.10), known to have significant gastrointestinal side effects [68]. Encapsulation and controlled release of IMC help mitigate these adverse effects. Composites of PLGA with *Nigella sativa* oil (NSO) or *Echium* oil (EQ) were prepared by the standard emulsion evaporation method, yielding 160 nm particles. The presence of hydrophobic oils allowed for a higher encapsulation efficiency of the lipophilic IMC compared to conventional PLGA (Table 16).

**TABLE 16:** Vegetable oil ratio of the nanocomposites, and their encapsulation efficiency of IMC. Adapted from J. Ghitman et al. [68].

Samples	PLGA/NSO ratio	PLGA/EQ ratio	Diameter [nm]	Encapsulation efficiency
PLGA‐IMC	0	0	165.2 ± 0.7	28.7 ± 2.4
HPON‐IMC	1/1	0	160.9 ± 1.9	61.4 ± 2.6
HPOE‐IMC	0	1/0.5	167.1 ± 1.8	37.7 ± 1.9

**FIGURE 18:**
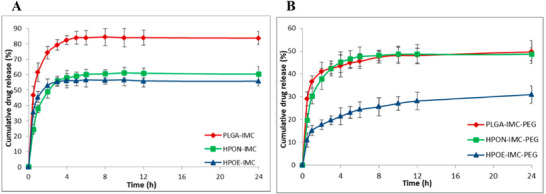
In vitro release profiles of IMC from control PLGA nanoparticles and PLGA‐VO nanocomposites (A) and PEG‐coated particles (B), in PBS at 37°C. Reproduced from J. Ghitman et al. from Ref [68], under CC BY 4.0 (MDPI).

Control PLGA particles released IMC with an initial burst of 46.7% release in the first 30 min, followed by a slower release phase that reached 83% after 24 h, and remained constant for the next 72 h (Figure 18A). The incorporation of VOs led to the same release profiles, but with a reduced initial burst: 24.4% for HPON‐IMC and 35.7% for HPOE‐IMC. This reduction is attributed to the hydrophobicity of the oils, which stabilizes IMC and limits water uptake from the release medium.

Moreover, post‐functionalization of the particle surfaces with PEG moieties further reduced the burst effect (Figure 18B). Acting as a diffusion barrier, the PEG coating made the burst release less pronounced, with 49.7%, 48.7% and 31.0% of IMC release during the first 24 h for PLGA‐IMC‐PEG, HPON‐IMC‐PEG, and HPOE‐IMC‐PEG, respectively. The release profiles of PEG‐coated PLGA nanoparticles and PEG‐coated nanocomposites containing NSO were similar, reaching an average of 57% and remaining constant thereafter. On the other hand, HPOE‐IMC‐PEG nanocomposites showed a much slower release without reaching a plateau.

Cell viability tests on fibroblast cells demonstrated the complete cytocompatibility of the nanocomposites without encapsulated IMC. In conclusion, these composites represent a first step toward the development of NSO‐ or EQ‐PLGA nanocomposites for the delivery of IMC. Building on these promising results, the same authors extended the concept of PLGA‐NSO composites to the encapsulation of multiple therapeutic compounds having different properties in terms of solubility and partition coefficients (Log P) [69]. Their encapsulation efficiencies and release kinetics were studied in PBS (pH = 7.4, 37°C) and are summarized in Table 17.

**TABLE 17:** Encapsulation efficiencies and release in 12 h (PBS, pH = 7.4, 37°C) of multiple molecules with different values of partition coefficients (Log P) [69].

Molecules	Log P	Encapsulation efficiency (%)	Release in 12 h (%)
(±) α‐tocopherol	10.00	61	0
Retinol	5.68	96	< 30
Curcumin	4.26	55	< 30
Indomethacin	3.10	64	61
Izohidrafural	2.10	82	32
Resveratrol	2.57	41	47
Hydrocortisone	1.72	20	58
Nitrofurantoin	‐ 0.47	< 10	—
5‐fluorouracil	‐ 0.89	< 10	—

As expected, lipophilic molecules exhibited higher encapsulation efficiencies in the nanocomposites, compared to hydrophilic compounds such as nitrofurantoin (Log P = ‐ 0.47) and 5‐fluorouracil (Log P = ‐ 0.89), which showed less than 10% of encapsulation. Consequently, their release kinetics were not studied. The differences in encapsulation efficiencies among the lipophilic compounds were attributed to specific structural interactions between the fatty acid contained in NSO and the therapeutic molecule structure. All release kinetics studied showed a burst effect in the first 30 min, followed by a sustained release over several days. For α‐tocopherol, its high lipophilicity prevented its release from the nanocarriers, due to strong interactions with the VO, which inhibited its diffusion into the PBS medium.

In conclusion, the hybridization of PLGA nanoparticles with VOs significantly improved the encapsulation efficiency of lipophilic therapeutic compounds and enabled their sustained release. The properties of the newly developed PONC are suitable for further development as drug delivery systems.

#### 4.1.2 Composites for Wound Dressings

Beyond their application as drug delivery systems, vegetable oil‐based polymers have also been explored for the development of functional biomaterials, such as wound healing films with intrinsic antibacterial properties. In this context, composites of a CO‐based polymer with zinc oxide‐chitosan nanoparticles were produced as wound healing films with antibacterial activity [70]. First, chitosan‐functionalized ZnO nanoparticles (CS‐ZnO) were dispersed in CO, before polymerization in a mold through the addition of the diisocyanate HDI and the cross‐linking catalyst glutaraldehyde. This process allowed the production of flexible films, with a Young's modulus ranging from 0.3 to 0.6 GPa, and elongations at break at around 80%. The incorporation of the nanoparticles did not greatly affect the mechanical properties.

**FIGURE 19:**
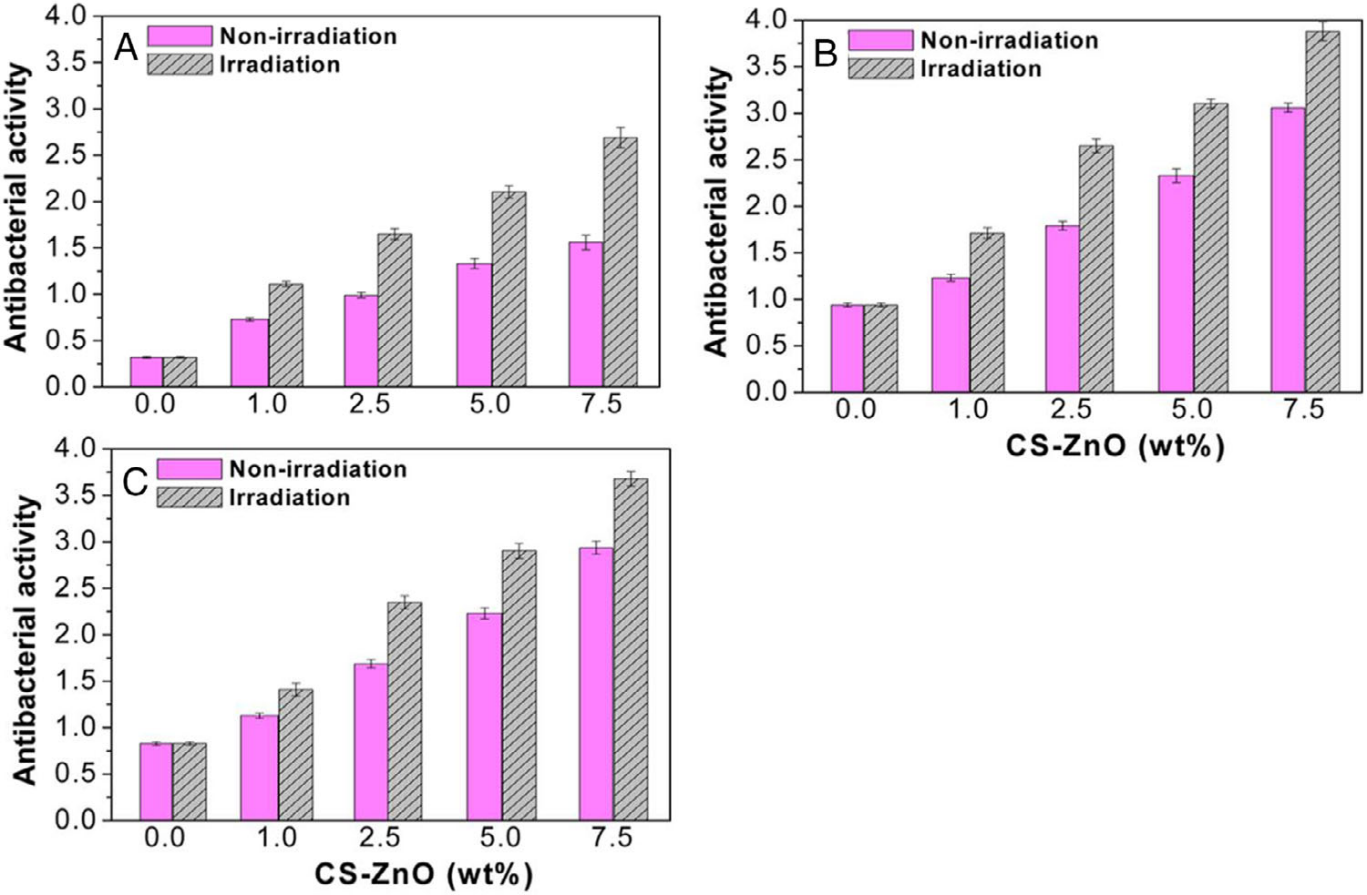
Antibacterial activities of CO/CS‐ZnO nanocomposite films against (a) E. coli (b) S. aureus (c) M. luteus, with or without UV irradiation. Reproduced with permission [70]. Copyright 2015, American Chemical Society.

ZnO‐chitosan grafted nanoparticles are known to have antimicrobial activities against both Gram‐positive and Gram‐negative bacteria, which can be enhanced by UV radiation [71]. Accordingly, the resulting films showed antibacterial activities against three bacteria strains (Figure 19). Moreover, cell viability assays revealed no significant cytotoxicity toward NHDF fibroblast cells.

**FIGURE 20:**
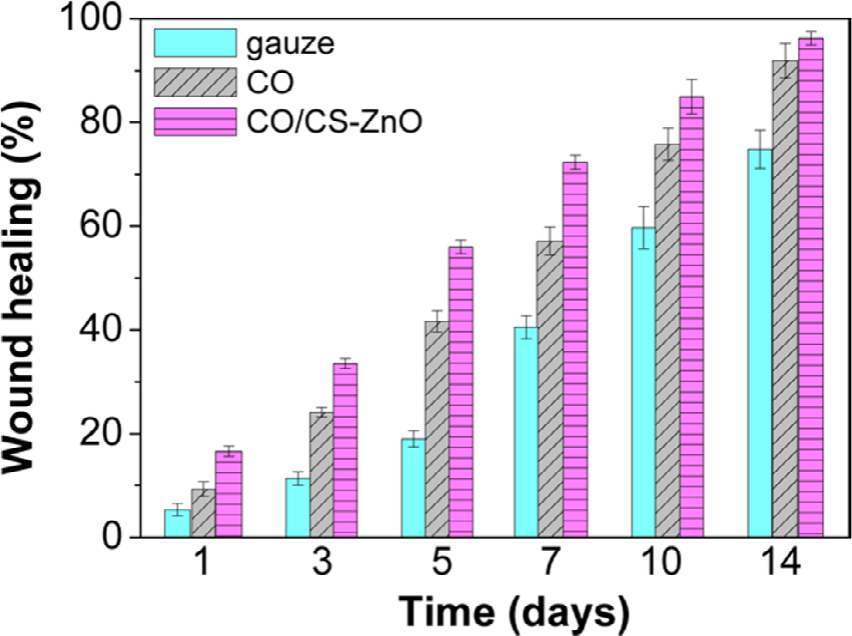
Wound healing as a function of time for CO/CS‐ZnO (5.0 wt.%) composites, CO‐based polyurethane, and gauze. Reproduced with permission [70]. Copyright 2015, American Chemical Society.

In vivo studies evaluating the wound healing effects of the composites were performed by applying the films to wounds in rats. The healing process was accelerated by the films, both with and without CS‐ZnO particles, compared to standard gauze, especially during the first week (Figure 20). During that period, significant differences were observed between the two film types: CO/CS‐ZnO films promoted faster healing, reaching 56% wound closure by day 5, compared to 42% for the CO film without nanoparticles. These results demonstrate that the incorporation of CS‐ZnO nanoparticles into CO‐based polyurethane matrices enabled the production of flexible films with both antibacterial activity and enhanced wound healing properties.

### 4.2 Oleogels

Organogels are gels formed by non‐polar molecules, including vegetable oils. Often called oleogels, they are prepared by the incorporation of structuring or gelling agents, called organogelators. These systems can form materials with a wide range of textures, from spreadable to elastic or brittle, depending on the oil/organogelators ratio [72]. Thus, they are of interest as lipophilic drug delivery systems.

**FIGURE 21:**
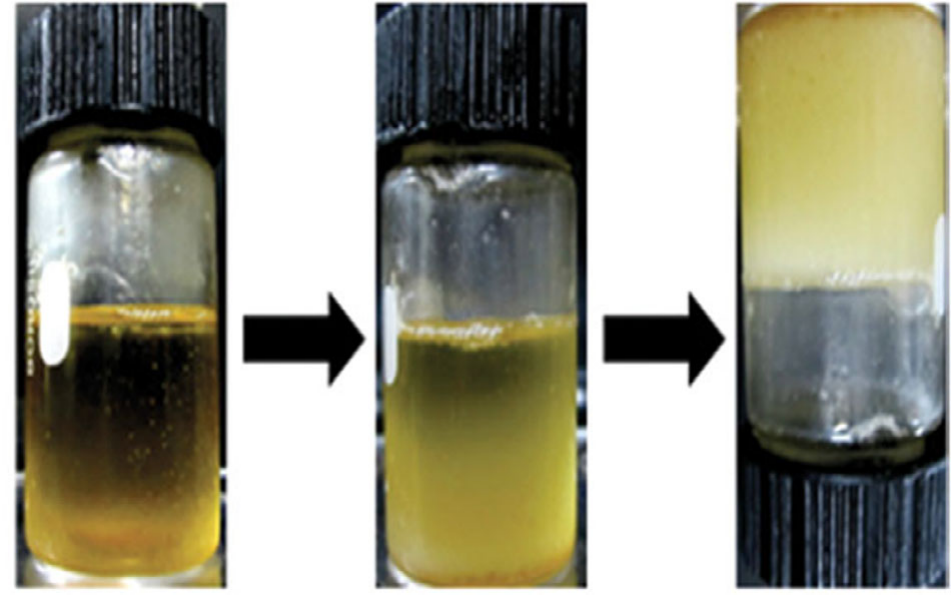
Gelation process and vial inversion method. Reproduced with permission [73]. Copyright 2012, Wiley.

Organogels prepared from olive oil, CO, and sesame oil with sorbitan monopalmitate or sorbitan monostearate as organogelators have been studied for this purpose. The gels were obtained by dissolving the organogelator in the VO at 70°C, followed by cooling down to room temperature once it was completely dissolved. They are considered gels when they stick to the bottom of the vial upon inversion (Figure 21).

Such a system was used with metronidazole (Log p = 0.75). This antibiotic molecule was dissolved in the oil prior to gelation to prepare drug‐loaded organogels [73]. These gels were found to be thermally stable and spreadable, and exhibited a slow release of the encapsulated drug in a Franz diffusion cell, while preserving its antimicrobial activity. Consequently, these olive oil‐based organogels can be used for topical drug delivery. Other organogels prepared with different VOs and organogelators also demonstrated sustained drug release (Table 18). Moreover, oleogels from safflower oil and N‐stearoyl‐L‐alanine methyl ester (SAM) as organogelator were developed for the formulation of rivastigmine (Log p = 2.3), a molecule clinically used in the treatment of Alzheimer's disease symptoms.

**TABLE 18:** Organogels from VOs developed as drug delivery systems.

Vegetable oil	Organogelator	Molecules	Release in 12 h	Reference
Olive oil (80%)	SMP (20%)	Metronidazole	26%	[73]
Olive oil (80%)	SMS (20%)	Metronidazole	39%	[73]
Castor oil (75%)	SMP (25%)	Metronidazole	28%	[74]
Castor oil (75%)	SMP (25%)	Metronidazole	84%	[74]
Sesame oil (85%)	SMS (15%)	Metronidazole	64%	[75]
Safflower oil (90%)	SAM (10%)	Rivastigmine	70%–90%	[76]

SMP = Sorbitan monopalmitate, SMS = Sorbitan monostearate, SAM = N‐stearoyl‐L‐Alanine methyl ester

Bigels have also been described, combining oleogels and hydrogels, where the organogel is dispersed as an emulsion within the hydrogel matrix. These systems are prepared by the incorporation of an organogel into the hydrogel at 70°C under stirring, before cooling to room temperature. They are formed without the use of surfactants or emulsifiers, and remain stable thanks to the gel network, which prevents phase separation. As a result, bigels exhibit mechanical properties that differ from those of the individual gel components, and they can be used as drug delivery systems for hydrophilic and lipophilic molecules, for transdermal administration (Table 19).

**TABLE 19:** Bigels developed as drug delivery systems.

Oleogel, organogelator	Hydrogel	Bioactive compound	Release	Reference
Sesame Oil, SMS	Guar gum	Ciprofloxacin	Up to 40% in 12h	[77]
Sunflower oil, SMP	Acacia gum	Metronidazole	87.72% in 12h	[78]
Sunflower oil, SMP	Guar gum	Metronidazole	75.75% in 12h	[78]
Sunflower oil, SMP	Xanthan gum	Metronidazole	73.04% in 12h	[78]
Sunflower oil, SMP	PVA	Metronidazole	Up to 51.97% in 12h	[79]
Sunflower oil, SMP	PVP	Metronidazole	Up to 63.65% in 12h	[79]
Sunflower oil, SMP	Sodium alginate	Metronidazole	71.68% in 12h	[79, 80]
Sunflower oil, SMP	Potato starch	Metronidazole	65.67% in 12h	[79, 80]
Sesame oil, SMS	Chitosan	Tenofovir	90% in 24h	[81]
Sesame oil, SMS	Pectin	Tenofovir	90% in 24h	[81]
Sesame oil, SMS	HPMC	Tenofovir	90% in 24h	[81]
Soybean oil, stearic acid	Gelatin	Ciprofloxacin	40% in 9h	[82]
Soybean oil, SMS	HPMC	Diltiazem HCl	49.4% in 6h	[83]

SMP = Sorbitan monopalmitate; SMS = Sorbitan monostearate; PVA = poly(vinyl alcohol); PVP = poly(vinylpyrrolidone); HPMC = hydroxypropyl methylcellulose

The production of oleogels and bigels allowed the encapsulation of both lipophilic and hydrophilic bioactive compounds through a relatively easy process. However, the release rate remains difficult to tune, with most formulations exhibiting a fast release profile. Therefore, depending on the intended application, this kind of formulation may not always be suitable.

## 5 Conclusions

Triglycerides, the main component of VOs, can be readily functionalized and chemically modified to produce polymers, such as polyester, polyurethanes, or hybrid materials. These triglyceride‐based polymers have been used as drug delivery systems, allowing the encapsulation and slow release of lipophilic therapeutic compounds. They have also been employed in the development of wound dressings, providing flexibility to the material, and functionalization with antimicrobial moieties has been achieved. Although numerous studies on this topic have been published and reviewed here, research is still ongoing to better control the release profiles of active compounds and to improve the biodegradability of these materials. In addition, efforts are being made to develop novel polymerization techniques that avoid the use of toxic catalysts in order to improve the cytocompatibility of the resulting materials.

## Author Contributions

The manuscript was written through the contributions of all authors. All authors have given approval to the final version of the manuscript.

## Conflicts of Interest

The authors declare no conflicts of interest.

## Data Availability

The authors have nothing to report.
